# Targeting KPC-Producing *Klebsiella pneumoniae* Biofilms: Genomic Profiles, Clinical Outcomes and Novel Combinations

**DOI:** 10.3390/microorganisms14081790

**Published:** 2026-08-14

**Authors:** Chiara Papalini, Giulia Bicchieraro, Luigi Tordelli Ruda, Alicia Yoke Wei Wong, Claudia Battistelli, Giovanni Genga, Andrea Tommasi, Anna Gidari, Daniela Francisci, Giuseppe Vittorio De Socio, Roberta Spaccapelo, Donatella Pietrella, Antonella Mencacci, Claudia Monari, Samuele Sabbatini

**Affiliations:** 1 Department of Medicine and Surgery, Section of Infectious Diseases, University of Perugia, 06129 Perugia, Italy; chiara.papalini@dottorandi.unipg.it (C.P.); andrea.tommasi@dottorandi.unipg.it (A.T.); daniela.francisci@unipg.it (D.F.); giuseppe.desocio@unipg.it (G.V.D.S.); 2 Infectious Diseases Clinic, Santa Maria della Misericordia Hospital, 06129 Perugia, Italy; anna.gidari@ospedale.perugia.it; 3 Department of Medicine and Surgery and Center of Functional Genomics (C.U.R.Ge.F), University of Perugia, 06129 Perugia, Italy; giulia.bicchieraro@unipg.it (G.B.); aliciayokewei.wong@unipg.it (A.Y.W.W.); roberta.spaccapelo@unipg.it (R.S.); 4 Department of Medicine and Surgery, Medical Microbiology Section, University of Perugia, 06129 Perugia, Italy; luigi.tordelliruda@dottorandi.unipg.it (L.T.R.); bclaudiab22@gmail.com (C.B.); donatella.pietrella@unipg.it (D.P.); antonella.mencacci@unipg.it (A.M.); 5 Infectious Diseases Clinic, Santa Maria Hospital, 05100 Terni, Italy; giovanni.genga@aospterni.it; 6 Respiratory Infectious Diseases Unit, National Institute for Infectious Diseases “Lazzaro Spallanzani” IRCCS, 00149 Rome, Italy; 7 Department of Life Sciences, Health and Health Professions, Link Campus University, 00165 Rome, Italy

**Keywords:** *Klebsiella pneumoniae* KPC, antibiotic resistance, beta lactam/beta lactamase inhibitor, biofilm, synergy test, catheter-related bloodstream infections

## Abstract

New beta-lactam/beta-lactamase inhibitor combinations (BL/BLICs) are licensed for *Klebsiella pneumoniae* carbapenemase (KPC)-producing *Klebsiella pneumoniae* (KPC-Kp) infections. However, data regarding their efficacy against bacterial biofilms and the potential of synergistic combinations remain limited. This study evaluated their efficacy on planktonic and biofilm forms of KPC-Kp isolated from catheter-related bloodstream infections (CRBSIs), correlating findings with genomic and clinical data. Twelve KPC-3-harboring isolates (ST512, *n* = 7; ST307, *n* = 3; ST101, *n* = 2) were characterized via whole-genome sequencing. Ceftazidime/avibactam (CAZ/AVI), meropenem/vaborbactam (MEM/VAB), and imipenem/relebactam (IMI/REL) were tested alone or combined with fosfomycin (FOS), cefepime (FEP), meropenem (MEM), or imipenem (IMI) using checkerboard assays. All BL/BLICs alone showed significant, comparable efficacy against biofilm-producing strains. However, combination testing revealed heterogeneous synergistic profiles. CAZ/AVI combined with MEM, IMI, or FEP achieved 100% synergy in both planktonic and biofilm states. MEM/VAB-based combinations demonstrated consistent synergy (50–100%), whereas IMI/REL showed higher synergistic rates in biofilm than in planktonic forms. Conversely, FOS-based regimens were significantly less effective against biofilms. These findings provide a clinical rationale for selecting optimized dual regimens to eradicate biofilms, offering a critical therapeutic strategy to preserve vascular devices when catheter removal is unfeasible.

## 1. Introduction

The 2023 ECDC epidemiological report emphasized the increasing incidence of bloodstream infections caused by carbapenem-resistant *Klebsiella pneumoniae* (CR-Kp) in Europe, citing a rate 57.5% higher than in 2019. Italy is one of the most affected nations, with CR-Kp representing between 25% and 50% of all invasive isolates [1]. The production of antibiotic-hydrolyzing enzymes is only one of the mechanisms adopted by *K. pneumoniae* to avoid antimicrobial effects. Another of its defense strategies is to build a protective heterogeneous structure called biofilm, constituted by a community of bacteria enclosed by extracellular polysaccharides, proteins and DNA. The biofilm adheres to biotic and abiotic surfaces including medical devices and is considered the main virulence factor responsible for healthcare-associated infections (HAIs) [2].

Within the biofilm architecture, the failure of antimicrobial therapies is driven by two distinct yet interconnected phenomena: resistance and tolerance. While the elements of antimicrobial resistance are genetically determined and inheritable, such as the production of carbapenemases or porin mutations, antimicrobial tolerance is a transient, phenotypic state of survival. Biofilm-associated tolerance is primarily mediated by the physical barrier of the extracellular polymeric substance (EPS) matrix, which restricts drug diffusion, and by the presence of a metabolic gradient. Deep within the biofilm layers, oxygen and nutrient deprivation lead to the formation of slow-growing or dormant sub-populations, known as persister cells, which are phenotypically immune to drugs targeting active bacterial metabolism.

These specific mechanisms pose unique therapeutic challenges in the management of catheter-related bloodstream infections (CRBSIs) caused by *Klebsiella pneumoniae* carbapenemase (KPC)-producing *Klebsiella pneumoniae* (KPC-Kp). In these clinical scenarios, the combination of genetic resistance and biofilm-mediated tolerance allows the bacteria to withstand antibiotic concentrations up to 1000-fold higher than their planktonic counterparts. Consequently, conventional monotherapies often fail to eradicate the embedded pathogen, turning the vascular device into a persistent reservoir that continuously seeds bacteria into the bloodstream and drives recurrent bacteremia unless the device is removed.

In the biennium 2022–2023, the prevalence of patients with at least one HAI in Europe was 7.1%, corresponding to 4.8 million HAIs per year. *Klebsiella* spp. is the second most frequent cause, and bloodstream infections account for 11.9% of all the HAIs [3]. It has been estimated that HAIs constitute 71% of cases of infections with antibiotic-resistant bacteria, including carbapenem-resistant Enterobacterales [4].

To offer a therapeutic chance to treat CR-Kp, in the last decade new beta lactam/beta lactamase inhibitor combinations (BL/BLICs), such as ceftazidime/avibactam (CAZ/AVI), meropenem/vaborbactam (MEM/VAB) and imipenem/relebactam (IMI/REL), have been licensed, but have encountered some resistances which have been previously described [5,6,7,8,9,10,11].

Although the clinical efficacy of new beta-lactam/beta-lactamase inhibitor combinations (BL/BLICs) against planktonic KPC-Kp is well documented, data regarding their performance against bacterial biofilms remain substantially scarce. This lack of evidence is particularly critical given the high genetic plasticity of KPC-positive clones under antibiotic selective pressure. While the international literature heavily focuses on the mutational dynamics and resistance emergence in *blaKPC-2*-bearing strains [12], the specific evolutionary trajectories of *blaKPC-3*-harboring clones (such as ST512 and ST307)—which frequently switch to the CAZ/AVI-resistant KPC-33 variant—require deeper investigation [13]. Conversely, comparative evidence evaluating newer combinations, such as meropenem/vaborbactam (MEM/VAB) and imipenem/relebactam (IMI/REL), against KPC-Kp biofilms is critically lacking, with few data available [14,15]. Furthermore, while dual-regimen combinations are frequently employed in clinical practice to prevent resistance emergence, their synergistic or antagonistic dynamics within the architecture of a biofilm remain largely unmapped. This significant knowledge gap limits the optimization of targeted therapies for device-associated infections, such as CRBSIs, with biofilm-mediated persistence as the primary driver of therapeutic failure. Therefore, the aim of this study was to analyze their effect on the planktonic form and biofilm of KPC-Kp both alone and in combination with fosfomycin (FOS), meropenem (MEM), imipenem (IMI) and cefepime (FEP). Crucially, these microbiological findings were correlated with genomic profiles obtained via whole-genome sequencing and retrospective clinical data from patients with CRBSIs, providing a comprehensive framework to optimize targeted therapeutic strategies.

## 2. Materials and Methods

### 2.1. Patient and Sample Characteristics

Twelve KPC-Kp clinical isolates were collected from all catheter-related bloodstream infections (CRBSIs) that occurred in Santa Maria della Misericordia Hospital of Perugia, Italy in 2023. Blood cultures were collected from patients with symptoms/signs of systemic infection and processed as described in a previous study [16]. Antimicrobial susceptibility testing was performed by reference broth microdilution using commercial lyophilized plates (Merlin Diagnostika GmBH, Bornheim-Hersel, Germany), and the results were interpreted according to European Committee on Antimicrobial Susceptibility Testing (EUCAST) clinical breakpoints [17]. Carbapenemase gene detection was carried out by the RT-PCR-based Xpert Carba-R test (Cepheid Inc., Sunnyvale, CA, USA). Isolates were stored at −80 °C until used. For each episode of CRBSI, the following information was collected: gender, age, co-morbidities, Sequential Organ Failure Assessment (SOFA) score, quick SOFA (qSOFA) score, implanted devices, surgery in the previous 30 days, KPC-Kp colonization at rectal swab, antibiotics administered in the previous 30 days, date of positive blood culture and duration of in-hospital stay before this date, ward where KPC-Kp was isolated, polymicrobial bacteremia, antimicrobial therapy and its duration and survival at 3, 14 and 30 days.

Recurrent CRBSI was defined as a positive blood culture from a specimen collected 4 days or more after the first infection [18].

### 2.2. Whole-Genome Sequencing (WGS)

Bacterial DNA was extracted using the QIAsymphony DSP Virus/Pathogen Kit (Qiagen, Hilden, Germany). Genomic libraries were prepared with the Illumina DNA Prep Kit and Nextera DNA CD Indexes (Illumina, San Diego, CA, USA). Quantification was performed using a Qubit™ Flex Fluorometer (Thermo Fisher Scientific, Waltham, MA, USA), and library quality was assessed with the Agilent 4150 TapeStation System and High Sensitivity DNA ScreenTape (Agilent, Santa Clara, CA, USA). Sequencing was carried out on the Illumina MiSeq with MiSeq Reagent Kits v3 (600 cycles) using the 2 × 150 bp paired-end as recommended by the Illumina DNA Prep kit protocol. FASTQ files were analyzed using services and analysis tools provided by the Center for Genomic Epidemiology (CGE) and Galaxy Europe web-based platform (https://usegalaxy.eu/, accessed on 11 August 2024) [19]. Species-level classification was confirmed via the DRAGEN (Dynamics Read Analysis for GENomics) Metagenomics Pipeline (https://sapac.illumina.com/products/by-type/informatics-products/basespace-sequence-hub/apps/dragen-metagenomics-pipeline.html, accessed on 29 August 2025) [20] on the Illumina BaseSpace Sequence Hub, using Kraken2 PlusPFP-8 (17 May 2021) as the reference database. The sequence type (ST) of the isolates was determined with multi-locus sequence typing (MLST) tools referencing PubMLST.org. De novo assembly was generated using SPAdes (Galaxy v3.15.5) [21], and scaffolds were annotated with Prokka (Galaxy v1.14.6) [22]. Pan-genome analysis was conducted using Roary (Galaxy v3.13.0), including the reference genome *K. pneumoniae* HS11286 (GenBank: GCA_000240185.2). Genes present in 99% of the genomes were designated as core genes. Phylogenetic analysis: Maximum-likelihood trees were built with IQ-TREE (Galaxy v2.4.0) and visualized by iTOL [23], and pan-genome comparisons were visualized in Phandango (https://jameshadfield.github.io/phandango/#/ accessed on 6 July 2025). Plasmid detection was performed with PlasmidFinder v2.1 [24] and MOB-Typer (Galaxy v3.1.9 + galaxy1), while antimicrobial resistance genes and mutations were identified using ResFinder v4.6.0 [25]. Virulence factors were detected by ABRicate (Galaxy v1.0.1). The bacterial genome sequencing data generated and analyzed during the present study have been deposited in the National Center for Biotechnology Information Sequence Read Archive (SRA) under BioProject accession no. PRJNA1496878.

### 2.3. In Vitro Biofilm Formation and Strain Classification

Biofilm formation of all the isolates was assessed as described previously [16]. In brief, 200 µL of bacterial cultures containing 1 × 10^7^ cells/mL n Mueller–Hinton broth were seeded into 96-well polystyrene flat-bottom plates (Corning Incorporated, Corning, NY, USA) and incubated for 72 h with or without medium renewal every 24 h. After incubation, plates were washed twice with saline solution, fixed with 99% methanol and stained for 15 min with 0.1% crystal violet (CV). Excess CV was rinsed twice with saline, and then 200 µL of 33% acetic acid was added and the biofilm was quantified using a microplate reader at 570 nm (Tecan Infinite M200; Tecan Trading AG, Männedorf, Switzerland). The results were used to classify KPC-Kp isolates into poor, weak, moderate and strong biofilm producers according to Devanga Ragupathi NK et al. [26].

### 2.4. Antibiotics and In Vitro Antimicrobial Susceptibility Testing

Ceftazidime/avibactam (Zavicefta^®^; Pfizer, Ringaskiddy, County Cork, Ireland), meropenem/vaborbactam (Vaborem^®^; Menarini International Operations, Luxembourg), imipenem/relebactam (Recarbrio^®^; Merck Sharp & Dohme B.V., Haarlem, The Netherland), fosfomycin (InfectoFos^®^; InfectoPharm, Heppenheim, Germany), meropenem (Venus Pharma GmbH, Werne, Germany), imipenem (Hikma Farmaceutica, Terrugem, Portugal) and cefepime (Qilu Pharma Spain, Madrid, Spain) were reconstituted according to the manufacturer’s instructions and aliquoted, and stock solutions were maintained at −80 °C until used. Minimum inhibitory concentrations (MICs) of all antibiotics used in this study were determined with the microdilution method according to EUCAST guidelines [27]. *Escherichia coli* ATCC 25922 was used as quality control. CAZ/AVI, MEM/VAB and IMI/REL were tested following EUCAST guidelines without maintaining a fixed concentration of beta-lactamase inhibitor, as commercially available antibiotics were serially diluted after reconstitution and then used for experiments.

### 2.5. Effect of Antibiotic Treatment on Strong-Biofilm-Producing Isolates

The eight isolates classified as strong-biofilm producers were grown for 48 h at 37 °C with medium renewal after 24 h of incubation as described above. Planktonic and loosely attached cells were removed by washing, and 200 µL of MH broth containing 5 different concentrations (1× to 16× the MICs previously obtained in vitro) of antibiotics CAZ/AVI, MEM/VAB and IMI/REL was added. The plates were incubated for 24 h in the same conditions, and biofilm biomass and metabolic activity were then quantified through crystal violet (CV) staining and the 2,3-bis-(2-methoxy-4-nitro-5-sulfophenyl)-2h-tetrazolium-5-carboxanilide (XTT, Invitrogen, Waltham, MA, USA) reduction assay, respectively. The XTT reduction assay was performed as previously described [26]. Biofilm-eradicating concentrations (BECs) of 50, 70 and 90 were defined as the concentrations of antibiotics able to reduce biofilm biomass or metabolic activity by 50, 70 or 90%, respectively, with respect to the control. BEC50, BEC70 and BEC90 were extrapolated using dose–response variable-slope (four parameters) non-linear regression.

### 2.6. Antibiotic Synergy Evaluation on Planktonic Bacteria

To evaluate antibiotic synergisms, the checkerboard assay was used. Bacterial inoculums were prepared at the final concentration of about 5 × 10^5^ cells/mL in cation-adjusted MH, and 200 µL was seeded into each well of a 96-well microtiter plate containing different concentrations of CAZ/AVI, MEM/VAB and IMI/REL (final concentration 0.03 − 2× MIC) in the rows and FOS, MEM, IMI and FEP (2-fold serial dilutions starting from the first concentration above the Cmax) in the columns. The plates were incubated for 24 h at 37 °C, and the fractional inhibitory concentration index (FICI) was evaluated in wells sited at the turbidity/non-turbidity interface of bacterial growth following the formula:

FICI = FIC_A_ + FIC_B_

where individual FIC values were obtained: FIC_A/B_ = (MIC of drug A/B in combination)/(MIC of drug A/B alone).

When the highest single antibiotic concentration tested was unable to inhibit bacterial growth, the concentration above the highest concentration tested was used for FICI calculation. Then, a FICI mean ≤ 0.5 was considered synergistic.

### 2.7. Antibiotic Synergy Evaluation on Strong-Biofilm-Producing Isolates

Mature biofilm of strong-biofilm-producing isolates was grown as described above, and the activity of antibiotic combinations was evaluated with a checkerboard assay. After biofilm washing, 200 µL of medium containing different concentrations of CAZ/AVI, MEM/VAB and IMI/REL (2-fold serial dilutions starting from the BEC50, BEC70 or BEC90) in the plate rows and FOS, MEM, IMI and FEP (2-fold serial dilutions starting from the first concentration above the Cmax) in the plate columns was added for 24 h at 37 °C. After incubation, the XTT reduction assay was performed as described above. For FICI calculation, the highest BEC achieved for each strain was used. When the highest single antibiotic concentration tested was unable to reach BEC50, BEC70 or BEC90, the concentration above the highest concentration tested was used for FICI calculation. A FICI mean ≤ 0.5 was considered synergistic. To overcome the inherent limitations of the fractional inhibitory concentration index (FICI), which relies on predefined thresholds and single-point MIC values, we employed SynergyFinder 3.0 for a more comprehensive assessment. Unlike the FICI, this multi-dimensional approach integrates the entire dose–response surface, accounting for sub-inhibitory interactions that are particularly relevant in the context of high-tolerance biofilm structures [28]. Thus, antibiotic combinations showing a FICI mean < 1 were selected for analysis with SynergyFinder software.

### 2.8. SynergyFinder Software

To mathematically evaluate drug interaction profiles, checkerboard data were analyzed using the SynergyFinder 3.0 software [28]. Synergy scores were calculated across the matrix using the highest single agent (HSA) reference model. According to the software parameters, a synergy score greater than 10 was considered significant for synergy, values between −10 and 10 indicated additive effects and scores less than −10 signified antagonism.

### 2.9. Statistical Analysis

Statistical analyses were performed using GraphPad Prism version 8. The normality of data distribution was assessed using the Kolmogorov–Smirnov test. For normally distributed data, a one-way ANOVA followed by Tukey’s post hoc test was used to perform multiple comparisons between the control group and the treated groups. For non-normally distributed data, the Kruskal–Wallis test followed by Dunn’s post hoc test was applied for pairwise comparisons. Data were presented as the mean with the respective standard deviation. A *p*-value < 0.05 was considered statistically significant.

## 3. Results

### 3.1. Clinical Data

In the established setting, 15 CRBSI episodes from 12 patients were positive for KPC-Kp. The experiments were driven by bacteria isolated during the first episode for each patient.

Patients’ characteristics are shown in Table 1.

Half of the patients were males, and the mean and median age were 65 and 66.5 years, respectively. Eleven out of twelve (92%) individuals carried other devices than the vascular one (eleven urinary catheters, four tracheostomy cannulas, two oro-tracheal intubations, two intra-cardiac devices, one intra-abdominal drainage), and five of twelve (42%) had undergone a surgical intervention in the previous 30 days (two cardiac and three abdominal operations). At CRBSI diagnosis, eight of twelve patients were known to be colonized by KPC-Kp, two were negative, one was colonized by ESBL-producing *K. pneumoniae* and one had not been tested. In the 30-day period before the positive blood culture, seven patients were administered with piperacillin/tazobactam, six with meropenem, three with ceftazidime/avibactam and one with cefiderocol, while two patients did not consume any antibiotic. Three CRBSIs (25%) were polymicrobial (one with *Enterococcus faecalis*, one *Candida albicans* and one *C. albicans* and *Enterococcus faecium*), and two were characterized by septic shock. Nine out of twelve patients (75%) underwent antimicrobial monotherapy. In addition, in all but four cases, the infected vascular device was removed. Thirty days after the bacteremia, five (42%) patients had died, two of them in the first 72 h. Four of the five deceased individuals corresponded to the four cases in which the device was kept in situ. Source control, indeed, represented the only statistically relevant discriminant between survivors and deceased patients (*p* = 0.010), instead of other parameters analyzed (gender, *p* = 0.621; mean age, *p* = 0.905; CCI >3, *p* = 0.293; intensive care unit, *p* = 0.576; oncologic–hematologic ward, *p* = 0.152; septic shock, *p* = 0.152; BL/BLIC resistance, *p* = 0.417; new BL/BLIC treatment, *p* = 1).

Table 2 shows the antimicrobial susceptibility pattern of the 12 isolates.

Interestingly, in two cases (patient n. 1 and 12), KPC-Kp isolated from recurred CRBSIs showed different antibiogram in comparison to the first infective episode. Bacteria isolated from patient n. 1, indeed, had acquired resistance to meropenem/vaborbactam (MIC 64), ceftazidime/avibactam (MIC 16/4) and fosfomycin (MIC > 128), while KPC-Kp of patient n. 12 in the second bacteremia had become resistant to imipenem/relebactam (MIC 4), ceftazidime/avibactam (MIC 16/4) and amikacin (MIC > 32).

### 3.2. Whole-Genome Sequencing (WGS)

**Figure 1:**
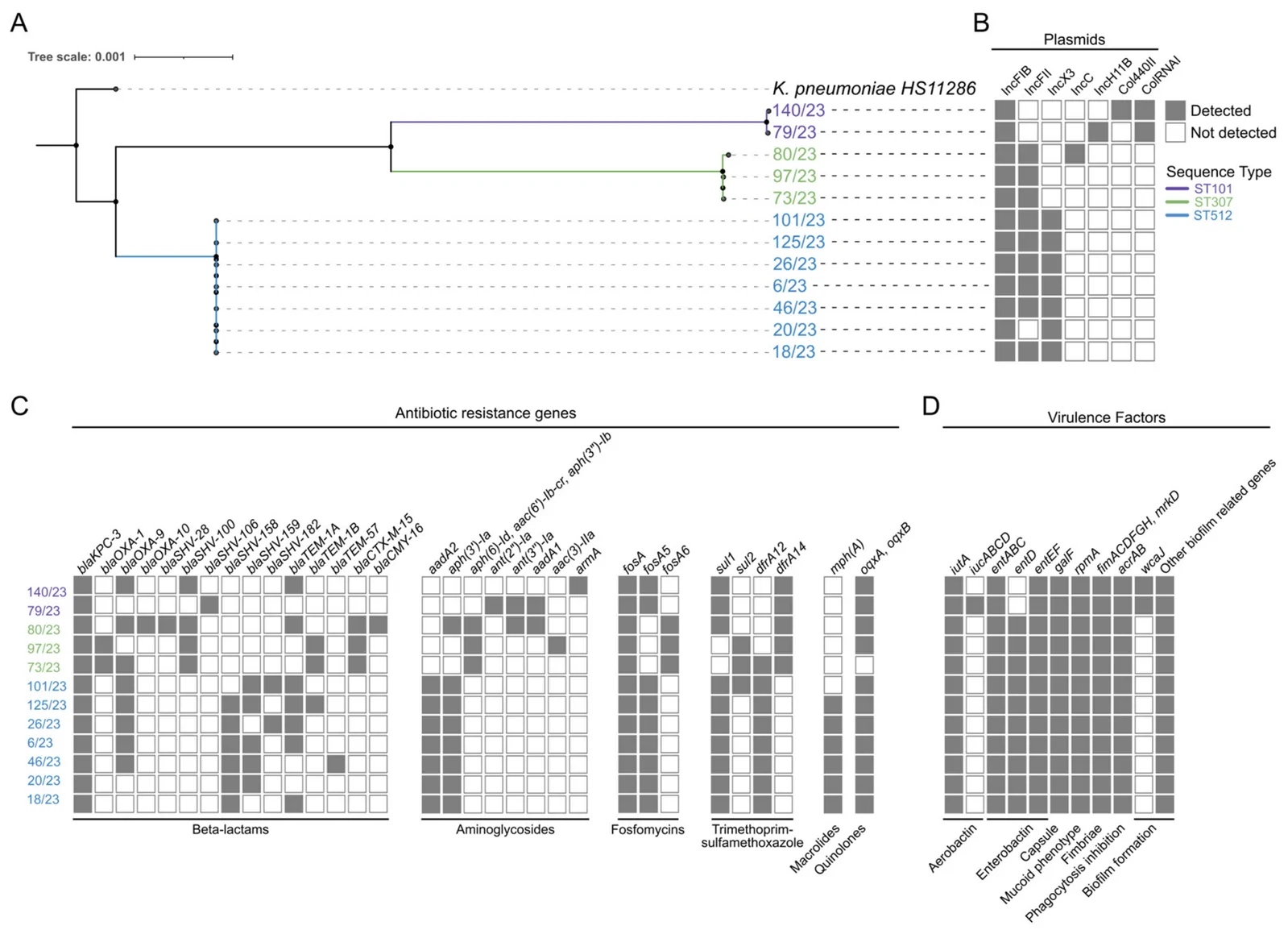
Genomic characterization of 12 *Klebsiella pneumoniae* isolates. (**A**) Core-genome phylogenetic tree showing clustering according to sequence type: ST101, ST307 and ST512. (**B**) Presence–absence matrix of plasmid replicons. (**C**) Distribution of antimicrobial resistance genes, grouped by antibiotic class. (**D**) Distribution of selected virulence-associated genes. Filled squares indicate gene or replicon detection, whereas empty squares indicate absence. Isolate labels are colored according to sequence type.

Whole-genome sequencing confirmed that all 12 isolates belonged to *Klebsiella pneumoniae*. Multi-locus sequence typing identified three lineages: ST512 (7/12, 58%), ST307 (3/12, 25%) and ST101 (2/12, 16.7%). Core-genome phylogeny showed clear clustering by sequence type (Figure 1A).

The ST512 isolates formed a highly compact clade, with limited genetic distances among isolates, consistent with clonal dissemination, whereas ST307 isolates constituted a genetically distinct lineage, in agreement with their emergence as a high-risk international clone. In contrast, ST101 isolates showed the greatest accessory genome diversity, consistent with increased genomic plasticity and adaptive potential. Plasmid replicon analysis revealed both conserved and sequence-type-associated profiles (Figure 1B), with IncFIB present in all isolates and IncFII in most isolates apart from lineage ST101 and sample 20/23. IncX3 was exclusively associated with ST512 and was detected in all seven isolates belonging to this lineage, whereas IncC, IncHI1B, ColRNAI and Col440II showed a more limited, isolate- or lineage-specific distribution (Figure 1B). All isolates carried several antibiotic resistance determinants (Figure 1C). The carbapenemase gene *bla*KPC-3 was detected in all genomes, consistent with resistance to carbapenems and other β-lactam agents. Additional β-lactamase genes varied according to sequence type and isolate. *bla*CTX-M-15 was restricted to ST307 isolates, while *bla*SHV-158 and *bla*SHV-159 were predominantly associated with ST512 isolates. Aminoglycoside-resistance genes also displayed lineage-associated patterns with *aadA2* and *aph*(*3′*)*-Ia* detected in all ST512 isolates. All isolates carried a *fosA*-type fosfomycin-resistance gene, with *fosA5* detected in ST101 and ST512 isolates, whereas ST307 isolates carried *fosA6*. Determinants associated with trimethoprim–sulfamethoxazole resistance were also common. Among them, *sul1* was detected in most isolates. A macrolide resistance gene, *mph*(*A*), was detected in most ST512 isolates, while quinolone-resistance genes, *oqxA* and *oqxB*, were detected in all samples except for 73/23. Virulence-associated genes involved in adhesion, biofilm formation, iron acquisition, capsule synthesis and efflux were broadly conserved, including *iutA*, *galF*, *rmpA*, *fimA-H*, *mrkD* and *acrAB* (Figure 1D). The enterobactin-encoding operon was complete in all isolates except for the ST101 isolates, which lack the *entD* gene. Among biofilm-related genes, *wcaJ* was identified only in the ST101 isolates. Details are shown in the Supplementary Materials (Figure S1, Tables S1–S5). No statistically relevant association was found between ST and septic shock (*p* = 0.152) or between ST and mortality (*p* = 0.558).

### 3.3. In Vitro Biofilm Formation

**Figure 2:**
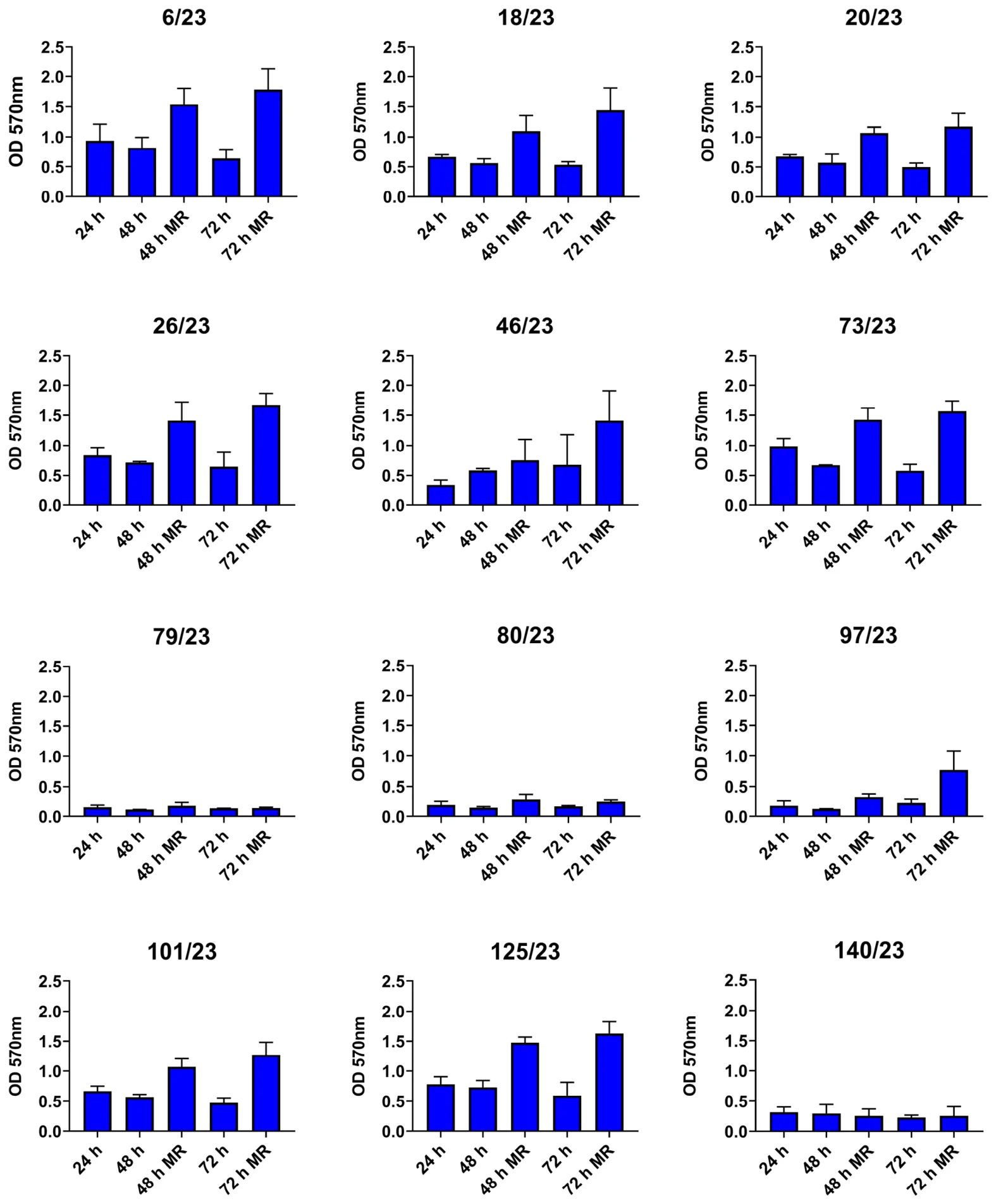
In vitro biofilm formation of *K. pneumoniae* clinical isolates. Clinical isolates were incubated for 24, 48 and 72 h with or without medium renewal (MR) every 24 h. Biofilm biomass was evaluated through crystal violet staining. Data are expressed as mean optical densities (OD) ±SD of at least three independent experiments with three technical replicates.

Maximum biofilm formation was observed after 48 h of incubation with medium renewal (Figure 2), with no significant further increase in biomass when extended to 72 h.

**Figure 3:**
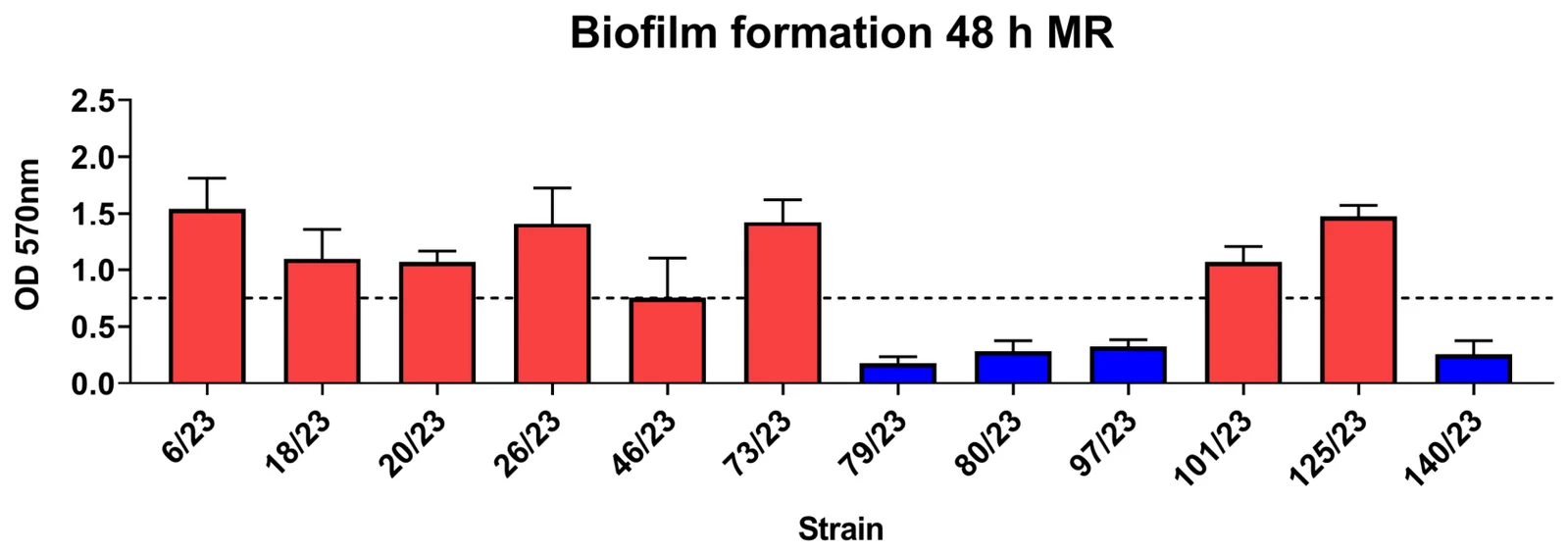
Classification of *K. pneumoniae* clinical isolates according to biofilm-forming ability. Mature 48 h-old biofilms were classified as poor-, weak-, moderate- and strong-biofilm producers according to Devanga Ragupathi NK et al. [26]. Red bars indicate strong-biofilm producers.

Furthermore, medium renewal was essential to avoid biofilm dispersal after 48 h of growth. Based on mature 48 h-old biofilm biomass, isolates were classified into poor-, weak-, moderate- and strong-biofilm-biomass producers. Eight out of twelve strains were able to produce a strong biofilm biomass (Figure 3). A statistically significant correlation was identified between biofilm production and ST (*p* = 0.01); however, no such association was found regarding septic shock (*p* = 0.091).

### 3.4. Antibiotic Treatment on Strong-Biofilm-Producing Isolates

CAZ/AVI, MEM/VAB and IMI/REL were tested in vitro to obtain MIC values, which are shown in Table 3.

**Figure 4:**
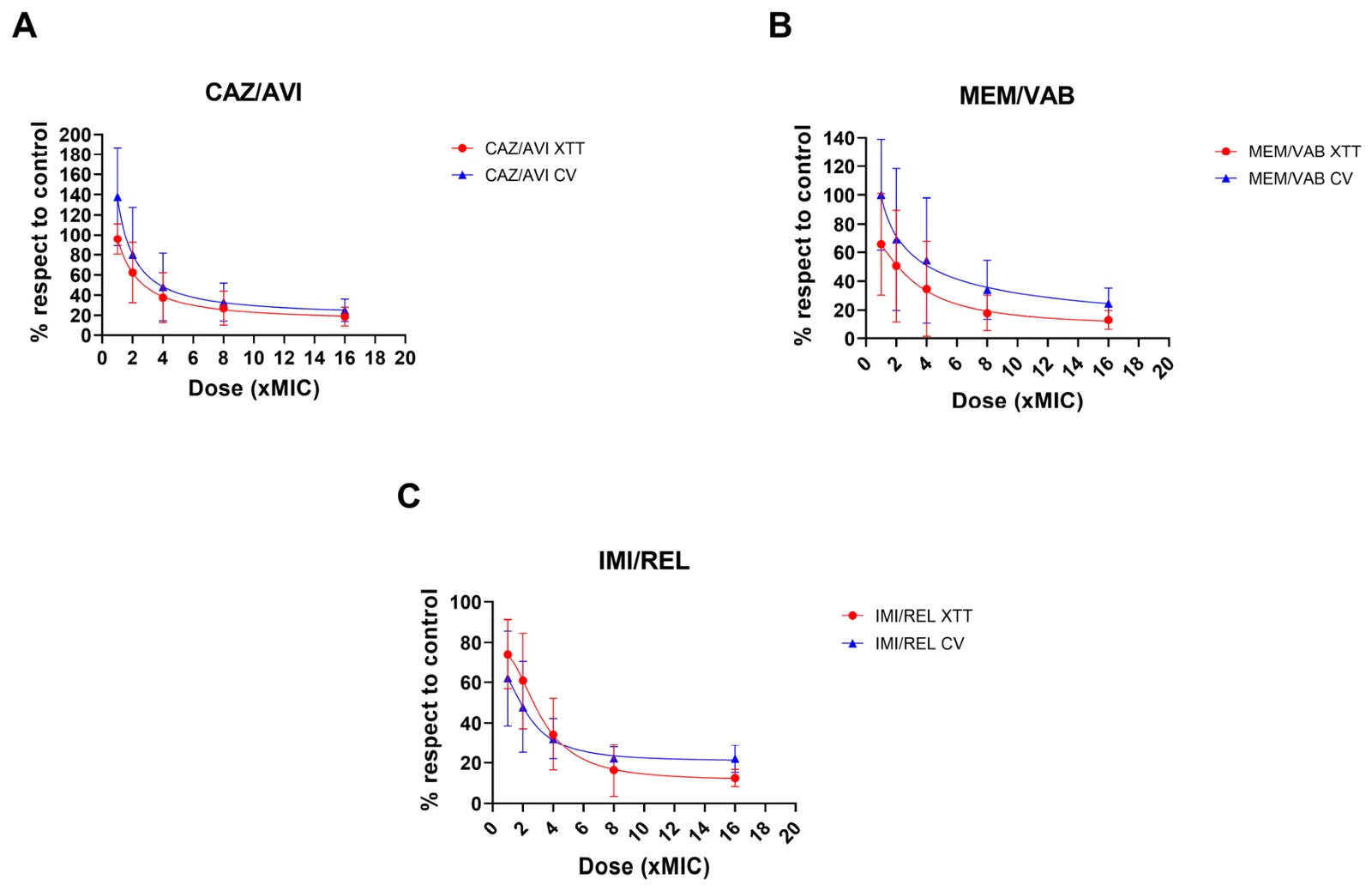
Impact of (**A**) CAZ/AVI, (**B**) MEM/VAB and (**C**) IMI/REL on eight strong-biofilm-producing *K. pneumoniae* clinical isolates. Mature 48 h biofilms were exposed to a range of antibiotic concentrations (1× to 16× MIC), followed by XTT and CV assays to evaluate metabolic activity and biomass, respectively. Data represent the mean percentage ± SD of metabolic activity (red lines) and biomass (blue lines) relative to untreated controls.

Then, mature biofilms of strong-biofilm-producing clinical isolates were treated with different concentrations of the three BL/BLICs (1× to 16× the MICs). Except for one isolate treated with MEM/VAB, all the BL/BLICs tested were able to significantly reduce biofilm metabolic activity and biomass at clinically meaningful concentrations (Figures S2–S4). Figure 4 shows the mean percentages of biofilm metabolic activity and biomass for the eight strong-biofilm-producing isolates following antibiotic treatment, compared to the untreated control.

At supra-MIC concentrations (8× and 16× MIC), the anti-biofilm effects of CAZ/AVI, MEM/VAB and IMI/REL converged, showing a markedly reduced standard deviation. This indicates that despite initial strain-to-strain variability at lower doses, high antibiotic concentrations exert a robust and highly consistent eradication effect across all strong-biofilm-producing isolates.

**Figure 5:**
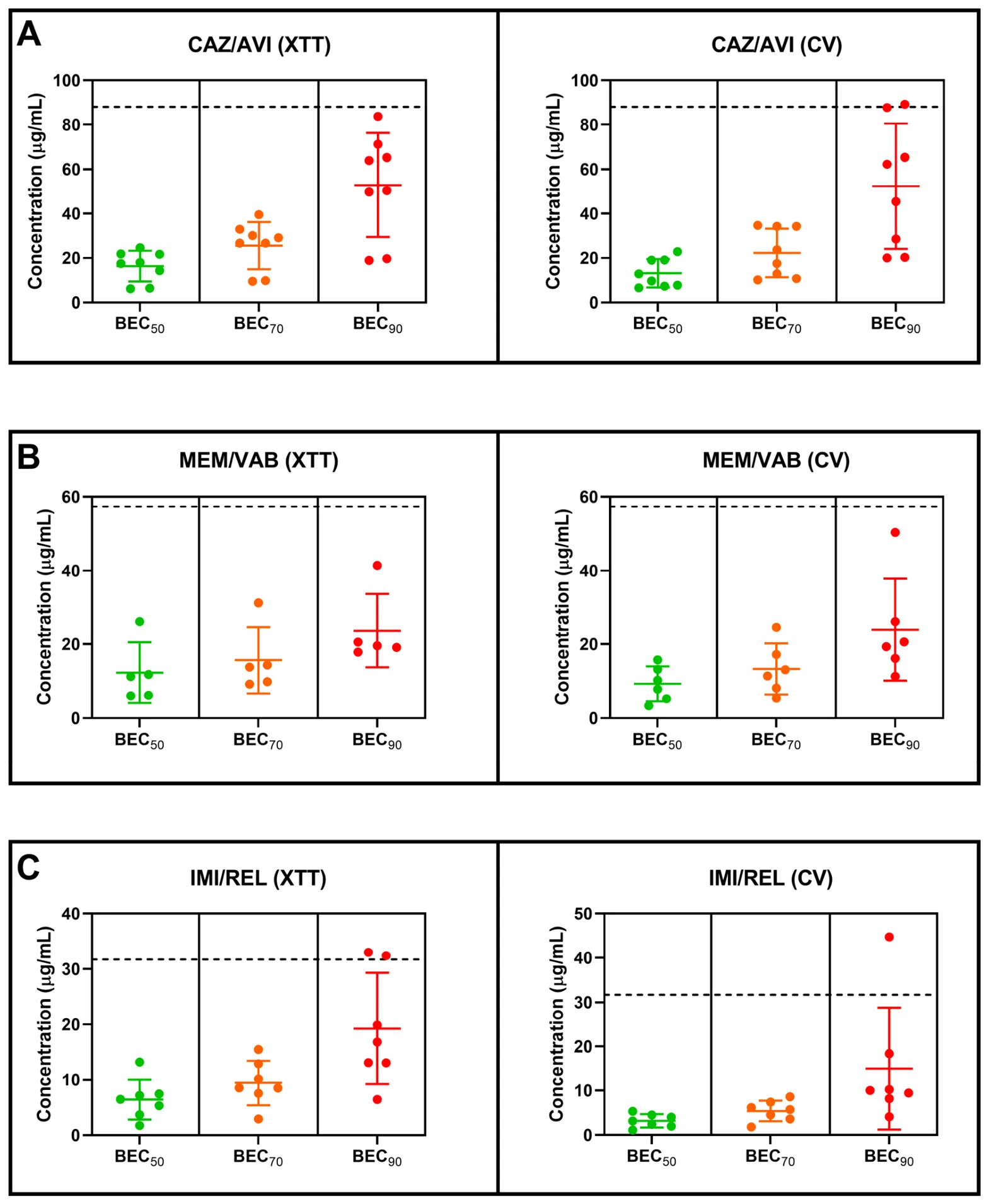
Biofilm-eradicating concentrations 50, 70 and 90 of antibiotic (**A**) CAZ/AVI, (**B**) MEM/VAB and (**C**) IMI/REL distribution. The concentrations of selected antibiotics able to reduce biofilm biomass or metabolic activity by 50, 70 or 90% compared to the control (BEC50, BEC70 and BEC90) were extrapolated from four-parameter dose–response curves. Data are expressed as mean ± SD of BEC50 (green), BEC70 (orange) and BEC90 (red) of strong-biofilm-producing clinical isolates. Ambiguous data were excluded from the plot. Dotted lines represent Cmax of each antibiotic.

The data in Figures S2–S4 were used to extrapolate BEC50, BEC70 and BEC90 by the dose–response variable-slope (four parameters) non-linear regression (Figures S5–S7). The obtained mean values of BECs for metabolic activity and biofilm biomass are presented in Figure 5.

The figure shows that the mean concentrations of antibiotics able to reduce both biofilm metabolic activity and biomass up to 90% compared to untreated controls were consistently below their reported mean peak serum concentrations (Cmax).

**Figure 6:**
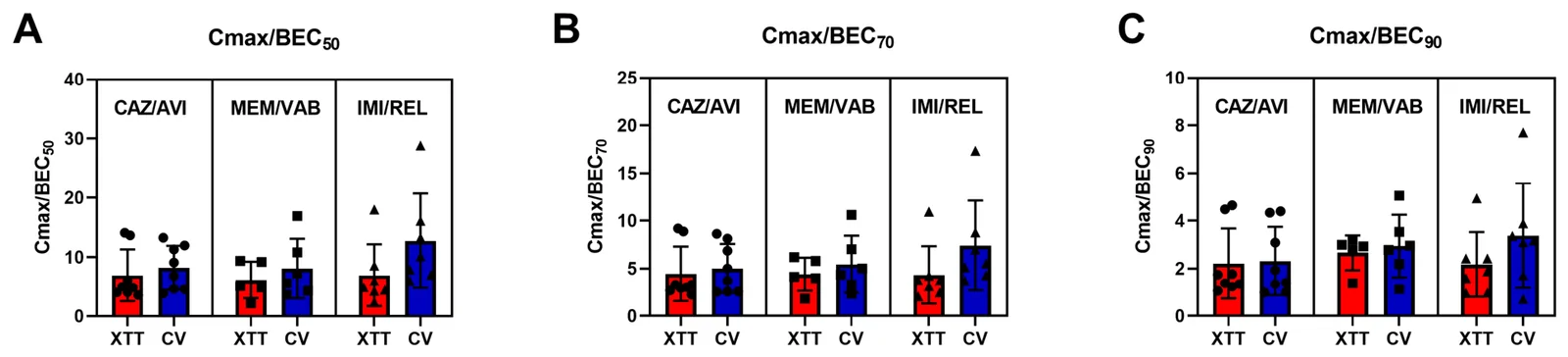
Comparison of antibiotic efficacy on *K. pneumoniae* mature biofilm. Ratios between maximum achievable serum concentration (Cmax) and (**A**) BEC50, (**B**) BEC70 or (**C**) BEC90 for biofilm biomass (blue bars) and metabolic activity (red bars) were used to compare the anti-biofilm activity of CAZ/AVI, MEM/VAB and IMI/REL antibiotics. Statistical significance among the different antibiotics across the BEC50, BEC70 and BEC90 thresholds was evaluated using a two-way repeated-measures ANOVA (Geisser–Greenhouse correction applied for sphericity), followed by Tukey’s post hoc multiple-comparison test. Data are expressed as mean ± SD of Cmax/BEC50, BEC70 or BEC90.

The comparison of the clinical potential of antibiotics was made using a Cmax/BEC ratio for every threshold considered (Figure 6).

No statistically significant differences were observed among the tested antibiotics (*p* > 0.05), demonstrating that CAZ/AVI, MEM/VAB and IMI/REL exhibited comparable efficacy profiles and maintained statistically overlapping Cmax/BEC ratios across all three evaluated thresholds (BEC50, BEC70 and BEC90).

### 3.5. Synergy on Planktonic Form of KPC-Kp Isolates

The combination of the three selected antibiotics with FOS, MEM, IMI or FEP was tested against the planktonic form of all *K. pneumoniae* isolates. The results of combinatory treatment are summarized in Table 4.

The lowest synergistic effect was observed for the combinations with FOS. IMI and MEM were synergistic against 100% of the strains if combined with CAZ/AVI, but only 50% and 8.3% of the isolates showed synergy when combined with MEM/VAB and IMI/REL, respectively. The highest synergy was found with FEP against planktonic KPC-Kp.

### 3.6. Synergy on Preformed Biofilm of KPC-Kp Isolates

The combinatory treatments were tested against mature KPC-Kp biofilms. Table 5 shows the results after FICI calculation. As expected, the synergistic effect of antibiotic combinations was not fully concordant with the results obtained against planktonic bacteria. Notably, IMI/REL + MEM showed an increase of synergistic effect.

Isolates showing FICI < 1 were further analyzed with SynergyFinder software. All the combinatory treatments showed synergistic activity against the totality of the isolates except for fosfomycin, which retained the lower effect as for planktonic KPC-Kp (Table 6).

All the 2D synergy landscape maps calculated using the ZIP reference model are shown in Figure S8. To pinpoint the exact drug ratios and concentrations responsible for the highest anti-biofilm efficacy, the most synergistic area (MSA) was extracted for each combination (Table 7).

This analysis revealed distinct, drug-specific concentration trends across the eight clinical isolates. The optimal synergistic concentrations for both CAZ/AVI and MEM/VAB combinations remained remarkably stable, displaying minimal variation regardless of the companion drug used. Specifically, the geometric mean of the MSA for CAZ/AVI stabilized at 2.59 µg/mL when combined with MEM or IMI, and at 2.83 µg/mL with FEP. Similarly, MEM/VAB showed highly consistent MSA geometric means of 1.54 µg/mL and 1.41 µg/mL with IMI and FEP, respectively. Notably, MEM/VAB achieved its peak synergistic landscape at concentrations nearly twofold lower than those required by CAZ/AVI, suggesting a higher intrinsic potency against the tested *K. pneumoniae* biofilms. On the other hand, IMI/REL combinations exhibited partner-dependent behavior. When paired with MEM, the geometric mean of the IMI/REL MSA concentration was 1.30 µg/mL. However, when combined with FEP, the required concentration of IMI/REL doubled, reaching a geometric mean of 2.59 µg/mL, although this increase did not reach statistical significance (*p* = 0.06).

## 4. Discussion

KPC-Kp HAIs are a silent plague, frequently involving medical devices and spreading widely in Italy. The advent of BL/BLICs has been welcomed as a breakthrough, but rising resistance and a lack of information about BL/BLICs’ efficacy against bacterial biofilm require further investigations.

Of our patients, all affected by KPC-Kp CRBSIs, nine of twelve were treated with the new antibiotics (four MEM/VAB and five CAZ/AVI), two individuals received older beta-lactams and one was only subjected to source control by removal of the infected device.

Whole-genome sequencing identified three KPC-3-producing *Klebsiella pneumoniae* lineages: ST512, ST307 and ST101. The predominance of ST512, the main clade detected in Italy, and the limited core-genome distances among these isolates support recent clonal transmission, likely within the healthcare setting. ST512 belongs to the internationally disseminated CG258 lineage and has frequently been associated with hospital outbreaks caused by KPC-producing *K. pneumoniae*. The uniform presence of IncX3 among the ST512 isolates, together with their closely related resistance profiles, further supports the expansion of a successful epidemic clone [29]. The detection of ST307 and ST101 was also epidemiologically relevant, as both are recognized high-risk lineages associated with hospital spread and multi-drug resistance. In this collection, ST307 isolates showed a characteristic resistance profile that included *bla*CTX-M-15, *aac*(*6′*)*-Ib-cr*, *aph*(*6*)*-Id*, *aph*(*3″*)*-Ib*, *fosA6* and *dfrA14*, suggesting that the successful dissemination of this lineage may be supported by resistance to multiple antimicrobial classes. ST101 was not present at a previous sequencing analysis of 2019 isolates in the same hospital [30].

The widespread detection of IncFIB and IncFII replicons indicates an important contribution of plasmids to the genomic adaptability of these isolates. IncF-type plasmids commonly carry antimicrobial-resistance and virulence determinants and may facilitate their maintenance and horizontal dissemination. Nevertheless, plasmid replicon typing alone cannot establish whether the detected resistance genes were carried by a specific plasmid or whether the plasmids were transferable. Complete plasmid reconstruction or long-read sequencing would therefore be required to determine the genomic location of *bla*KPC-3 and the contribution of individual plasmids to transmission [31,32].

The different STs were not significantly associated with specific hospital wards or mortality rate; however, a potential correlation with biofilm production was noted. Specifically, ST512 emerged as the overwhelmingly dominant and most aggressive biofilm-producing clone in our CRBSI setting. Conversely, ST101 and ST307 isolates were mostly classified as weak-biofilm producers, with the sole exception of ST307 isolate 73/23 (patient n. 6). This phenotypic pattern suggests that the closely related ST512 isolates may share genomic or regulatory characteristics that favor surface colonization and biofilm maturation. All isolates carried major adhesion-associated determinants, including the type 1 fimbrial genes *fimA*–*fimH* and the type 3 fimbrial adhesin gene *mrkD*. Therefore, the stronger-biofilm phenotype of ST512 cannot be explained simply by the presence or absence of these genes. The poor-biofilm phenotype of the two ST101 isolates may be partially consistent with their distinct capsule-associated profile. Both carried *wcaJ*, which is involved in a capsular polysaccharide synthesis and may influence biofilm architecture [33]. However, its role in biofilm development is complex, as abundant capsule production may also mask fimbrial adhesins and reduce initial attachment to biotic or abiotic surfaces. Recent experimental evidence has shown that modification or loss of capsule synthesis may enhance certain stages of biofilm adaptation, depending on the strain background [34]. Both ST101 isolates also lacked *entD*, suggesting an incomplete enterobactin biosynthesis pathway, except that isolate 79/23 carried the complete aerobactin locus *iucA*–*iucD*. Moreover, our observation is based on only two ST101 isolates, and therefore the presence of *wcaJ* and the absence of *entD* cannot be directly linked to the reduced biofilm-forming capacity found in these isolates. Additional studies involving a larger number of isolates and experiments are required to clarify the contribution of *wcaJ* and other lineage-specific determinants to the biofilm phenotype. ST307 isolates were also poor-biofilm producers despite carrying the conserved fimbrial determinants. Previous genomic studies have identified fimbrial, siderophore, capsular and plasmid-associated adaptive determinants in ST307, but biofilm formation can vary substantially among isolates belonging to this lineage [35]. Determination of ST could also influence the pattern of resistance [36]: we tested antimicrobial activity on the planktonic form of all 12 KPC-Kp, but the phenotypes were quite similar across the sequence types, and the major diversity arose for 79/23, which was resistant to IMI/REL and had the highest MIC for MEM/VAB. Notably, detected in this sample were *ant*(*2”*)*Ia* and *bla*SHV-106 resistance genes, potentially involved in this different antibiotic resistance profile [37]. The ST512 isolates bore the IncX3 plasmid and aminoglycoside and trimethoprim resistance genes, confirming the phenotype that has been observed from other KPC-producing *Klebsiella pneumoniae* ST512 in the literature [38]. Moreover, all isolates that belonged to ST307 harbored genes which conferred resistance to aminoglycoside, fosfomycin, sulfamethoxazole and beta-lactams, as observed by Patil et al. [39]. As reported by other authors [36,40], resistance to IMI/REL is mediated by disruption of the *ompK35* and *ompK36* porin-encoding genes and could be associated with MEM/VAB resistance as well. In our case, WGS found mutations of *ompK36* in all the isolates. They were nucleotide substitutions and were not associated with resistance to the new BL/BLICs, even if the literature demonstrated that mutations on this site could induce resistance to CAZ/AVI, MEM/VAB and IMI/REL [41,42]. Boattini et al. suggest that resistance to the new BL/BLICs could be also mediated by the co-presence of *bla*KPC genes and other beta-lactamase genes, such as *blaOXA*, *bla*SHV and *bla*CTX [43]. In this study, the isolates that showed low synergic effect bore both carbapenemase genes and beta-lactamase genes.

All antibiotics tested on strong-biofilm producers gave satisfying results, reaching BEC50, 70 and 90 by using drug concentrations usually inferior to the Cmax, except for MEM/VAB inactivity against the 73/23 isolate. This behavior is likely attributable to the exceptionally low MIC observed in this specific strain, which resulted in the use of sub-therapeutic concentrations during in vitro testing, falling below clinically relevant thresholds. In our study, we evaluated the concentrations of BL/BLICs able to reduce the metabolic activity and biomass of strong-biofilm-producing clinical isolates by 50, 70 and 90% compared to controls. As shown in Figure 6, the mean concentrations of all thresholds remained below the known mean maximum serum concentration (Cmax) of each antibiotic tested. Based on these findings, we evaluated the clinical potential of the three antibiotics by calculating the Cmax/BEC50, 70 and 90 ratios, balancing in vitro anti-biofilm activity with standard human pharmacokinetic parameters. Our statistical analysis revealed no significant differences among the Cmax/BEC50, 70 and 90 ratios of the tested drugs (*p* > 0.05). This suggests that all three antibiotics exhibit a comparable pharmacodynamic potential against *K. pneumoniae* biofilms. Consequently, from a purely PK/PD standpoint focused on initial biofilm eradication capability, none of the molecules emerged as superior. However, other clinical and biological factors, such as matrix penetration kinetics, local pH and the risk of resistance selection inside the biofilm, should be considered when choosing the optimal therapy. A recent article focusing on two isolates, one ST101 and one ST307, confirmed MEM/VAB in vitro efficacy on bacterial biofilm [14], while Birteksoz Tan et al. found greater efficacy of IMI/REL against biofilm of imipenem- and/or meropenem-resistant, metallo-beta-lactamase-negative *K. pneumoniae* clinical isolates [15]. No other studies, to our knowledge, have analyzed the selected BL/BLIC effect on this form of bacteria.

In light of the rapid development of resistance, we also tested other antibiotics likely to be associated with the new BL/BLICs. We focused on FOS due to its known anti-biofilm activity [44], carbapenems for their synergistic effects [16] and FEP because of its action on different penicillin-binding proteins (PBPs) [45]. Specifically, the selection of the partner antibiotic was guided by published evidence. Regarding carbapenems, previous studies have shown a synergistic effect from the combination of meropenem and ceftazidime/avibactam (MEM + CAZ/AVI), with clinical reports documenting improved outcomes [16,46,47,48]. Furthermore, prior to the introduction of BL/BLICs, several studies highlighted the benefits of double-carbapenem regimens in terms of bactericidal activity and patient survival [49,50,51,52,53]. On the other hand, testing cefepime (FEP) in combination with BL/BLICs was prompted by the renewed interest in this drug, which recently led to the commercialization of cefepime/enmetazobactam, a regimen suggested to play a role in managing certain *Klebsiella pneumoniae* KPC infections [54,55,56,57]. In this context, Crema et al. also discussed the potential co-administration of cefepime/enmetazobactam with MEM [58].

As expected, antimicrobial combination effects have been shown to be different in planktonic and biofilm forms. In planktonic form, FEP is the most promising partner drug, always synergistic with CAZ/AVI (100% of the strains) and against 83.3% of the strains with MEM/VAB and IMI/REL (both combinations are not synergistic against strains 18/23 and 20/23). Notably, the combinations showed a FICI mean for the strains 18/23 and 20/23 of 0.63 and 0.64 for MEM/VAB + FEP and 0.55 and 0.54 for IMI/REL + FEP, respectively. It is worth pointing out that in these two cases, MEM/VAB and IMI/REL demonstrated the highest effect alone, so that the association of a second antibiotic had a lower influence and clinical relevance. Nevertheless, it could be useful in case of resistance. The association of FEP with MEM/VAB or IMI/REL, in fact, showed a synergistic effect below Cmax doses, even for strains with the highest MIC. On the other hand, FOS and carbapenems were synergistic in fewer isolates. The marked resistance to fosfomycin was confirmed by the detection of the *fosA* gene by WGS [59].

Concerning the effect on biofilm, FICI and SynergyFinder gave slightly different results, showing more evident synergy with the second method. Unlike the traditional FICI method, which strictly constrains the evaluation to single BEC endpoints, the software-assisted surface response analysis allowed us to investigate the non-linear cooperative effects of the combinations across a wide range of concentrations, accounting for the inherent biological and physical complexity of the biofilm matrix.

Synergy analysis was performed using the highest single agent (HSA) model. This model was selected for its conservative and clinically relevant approach, as it defines synergy only when the combined effect exceeds the efficacy of the most potent individual drug at the same concentration. This ensures that the observed enhancement is not merely an additive effect but a true pharmacological potentiation, a crucial distinction when evaluating antibiotic resilience in biofilm structures. Both methods agreed on the poor contribution of FOS and the superior results of double-carbapenem or FEP association. These findings confirm the results of our previous experiments [16] but differ a bit from the checkerboard results on the planktonic form.

An interesting finding emerges from the analysis of the geometric mean concentrations within the most synergistic areas (MSAs). The optimal synergistic concentrations of CAZ/AVI and MEM/VAB remained remarkably consistent regardless of the companion drug. This consistency suggests a robust, partner-independent mechanistical synergy, likely driven by the potent and uniform inhibition of beta-lactamases within the biofilm matrix by avibactam and vaborbactam. Notably, MEM/VAB achieved maximum synergy at concentrations nearly twofold lower than CAZ/AVI, highlighting its high intrinsic anti-biofilm activity. In contrast, IMI/REL exhibited a distinct behavior, showing a low MSA mean concentration when combined with MEM, which doubled when paired with FEP. This discrepancy could be attributed to the differential penicillin-binding protein (PBP)-targeting profiles; while imipenem and meropenem cooperatively target PBP-2 and PBP-1, cefepime preferentially binds to PBP-3. The requirement for higher IMI/REL concentrations when combined with FEP might reflect the need for an enhanced outer-membrane or matrix penetration pressure to achieve a multi-PBP complementary blockade within the *K. pneumoniae* biofilm architecture.

The limited number of isolates tested makes it difficult to draw confident correlations between patients’ characteristics and in vitro experiments. In light of this, clinical records could not show any link between mortality and therapy prescribed, and the evidence points to source control as the strongest predictor of survival. The WGS, too, fails to offer an explanation to the different in vitro behavior of the tested strains, except for the possible role of *wcaJ*.

Beyond these clinical and genomic constraints, several methodological limitations must be considered when extrapolating our findings to clinical CRBSI scenarios. First, our experiments utilized a static polystyrene model, which lacks the hydrodynamic fluid flow and continuous nutrient replenishment found in the bloodstream. Shear stress is known to significantly alter biofilm thickness, density and metabolic activity. Second, biofilm architecture and matrix composition can vary quantitatively and qualitatively depending on the biomaterial surface, meaning that results obtained on polystyrene plates may not fully replicate those on medical-grade silicone or polyurethane catheters. Lastly, our testing was conducted in protein-free broth. In the clinical setting, the presence of serum proteins and host cellular components immediately covers the catheter surface with a conditioning film, which promotes bacterial attachment. Furthermore, serum protein binding reduces the fraction of free, active beta-lactams available to penetrate the biofilm.

In addition to these model-specific constraints, a methodological caveat must be considered regarding our MIC and BEC determinations for the novel BL/BLICs. Because we diluted the integrated commercial products rather than maintaining a fixed concentration of the inhibitors as per EUCAST guidelines, the concentration of BLICs decreased proportionally in the highest dilutions. While this choice aligns with real-world pharmacokinetic co-dilution principles, it might have led to a relative underestimation of susceptibility in specific strains heavily dependent on high, sustained inhibitor concentrations to overcome massive carbapenemase production.

A key strength of this study lies in the unique opportunity to qualitatively cross-reference our microbiological findings with the clinical outcomes of the 12 patients from whom the KPC-Kp strains were isolated (Table S6). Due to the small sample size, formal statistical correlation is not feasible; however, important clinical observations can be drawn. Notably, the clinical data demonstrate that source control via catheter removal was the dominant determinant of 30-day survival. All four patients for whom catheter removal were deemed clinically unfeasible died, even though their isolating strains did not exhibit higher biofilm-forming capacities or significantly higher BEC90 values compared to those of the survivors. On the other hand, a statistically significant difference was observed between surviving and deceased patients regarding the geometric mean planktonic MICs of CAZ/AVI and IMI/REL.

Consequently, while our dose–response curves and synergy matrices provide a robust screening baseline, these in vitro findings must be interpreted with caution and should be considered as promising therapeutic candidates requiring further validation in dynamic flow systems and in vivo animal models.

Nevertheless, completely removing the infected device is sometimes unfeasible or highly risky for the patient. In this scenario, where catheter salvage strategies become mandatory, our study addresses a critical knowledge gap regarding the anti-biofilm activity of novel BL/BLICs. By proposing optimized combination regimens, these findings offer a rational framework designed to counteract the rapid emergence of resistance often observed with monotherapy, providing valuable translationally oriented insights for refractory CRBSI management.

## 5. Conclusions

In conclusion, KPC-Kp remains a critical threat in healthcare-associated infections, particularly those involving medical devices. Our findings suggest a potential biofilm eradication capacity for all three new BL/BLICs within the evaluated cohort. Genomic analysis through WGS revealed that the evaluated high-risk clones ST101 and ST307, despite their rapid spread in Italy, behaved generally as weak-biofilm producers in vitro. The presence of the *wcaJ* gene in ST101, together with the lack of a complete enterobactin operon, may partially explain this reduced capacity, although this hypothesis warrants wider validation. Furthermore, while mutations in *ompK36* were ubiquitous, they did not consistently translate into high-level resistance to the new BL/BLICs in our cohort. Regarding combination therapies, FEP emerged as a highly promising partner, showing widespread synergy in both planktonic and biofilm forms, while the “double-carbapenem” approach also provided a superior in vitro effect compared to FOS. Crucially, while source control remains the gold standard for patient survival, these results provide a preliminary pharmacological rationale for catheter salvage strategies when removal is clinically unfeasible. Ultimately, rather than definitive therapeutic options, these in vitro data serve as a robust screening baseline, generating promising candidate regimens that strictly require prospective verification in physiologically relevant, dynamic flow models and in vivo clinical evaluations.

## Figures and Tables

**Table 1:** Patients’ characteristics.

Pt	Gender	Age	CCI	SOFA	qSOFA	Device	Ward	Therapy	Duration (Days)	Exitus	Relapse
1	F	47	0	1	0	CVC	I	MEM/VAB	10	Not	Yes
2	M	71	5	7	2	AC	I	Not	0	Not	Yes
3	M	42	0	NA	1	CVC	M	CAZ/AVI	16	Not	Not
4	M	66	2	5	2	CVC	I	MEM/VAB + FOS	NA	Not	Not
5	M	67	11	NA	1	PAC	O	PIP/TAZ + AMK	3	Yes	Not
6	F	66	6	NA	NA	CVC	M	MEM	14	Not	Not
7	F	73	6	14	2	CVC	O	CAZ/AVI	31	Yes	Not
8	M	62	3	6	1	AC	I	MEM/VAB	11	Yes	Not
9	F	80	6	NA	1	PICC	M	CAZ/AVI	16	Not	Not
10	M	51	6	NA	NA	PICC	M	CAZ/AVI	1	Yes	Not
11	F	78	6	6	2	CVC	M	MEM/VAB	16	Not	Not
12	F	73	5	NA	3	PAC	M	CAZ/AVI	13	Yes	Yes

Abbreviations: CCI, Charlson Comorbidity Index; SOFA, Sequential Organ Failure Assessment; qSOFA, quick Sequential Organ Failure Assessment; M, male; F, female; NA, not available; CVC, central venous catheter; AC, arterial catheter; PICC, peripherally inserted central catheter; PAC, port à cath; I, intensive care unit; O, oncological/hematologic ward; M, medical ward; MEM/VAB, meropenem/vaborbactam; CAZ/AVI, ceftazidime/avibactam; FOS, fosfomycin; PIP/TAZ, piperacillin/tazobactam; AMK, amikacin; MEM, meropenem.

**Table 2:** Antimicrobial susceptibility.

Pt	Isolate n.	ST	AMK	FDC	CAZ/AVI	CPX/LVX	COL	FOS	IMI	IMI/REL	MEM	MEM/VAB	TGC
1	6/23	512	≤4	R	2/4	>2	≤1	≤32	>8	0.25/4	32	0.5/8	≤0.25
2	18/23	512	≤4	NA	4/4	>2	≤1	≤32	>8	0.25/4	128	0.5/8	≤0.25
3	20/23	512	8	S	2/4	>2	≤1	≤32	>8	NA	4	0.04/8	1
4	26/23	512	≤4	R	2/4	>2	≤1	≤32	>8	0.25/4	32	0.25/8	≤0.25
5	46/23	512	≤4	NA	4/4	>2	≤1	>128	>8	0.5/4	128	0.5/8	≤0.25
6	73/23	307	≤4	S	2/4	>2	≤1	8	>8	NA	8	≤0.5/8	0.5
7	79/23	101	≤4	S	4/4	>2	≤1	>256	>8	4/4	>128	2/8	0.5
8	80/23	307	32	NA	4/4	>2	2	≤32	>8	0.5/4	64	≤0.5/8	0.5
9	97/23	307	≤4	0.5	2/4	>2	≤1	8	>8	≤0.25/4	16	≤0.5/8	≤0.25
10	101/23	512	≤4	1	8/4	>2	≤1	32	>8	0.5/4	>128	≤0.5/8	≤0.25
11	125/23	512	≤4	NA	4/4	>2	≤1	32	>8	≤0.25/4	32	≤0.5/8	≤0.25
12	140/23	101	8	S	4/4	>2	2	32	>8	1/4	128	≤0.5/8	1

Abbreviations: ST, sequence type; AMK, amikacin; FDC, cefiderocol; CAZ/AVI, ceftazidime/avibactam; CPX, ciprofloxacin; LVX, levofloxacin; COL, colistin; FOS, fosfomycin; IMI, imipenem; IMI/REL, imipenem/relebactam; MEM, meropenem; MEM/VAB, meropenem/vaborbactam; TGC, tigecycline; green shading indicates susceptibility; red shading indicates resistance.

**Table 3:** In vitro antimicrobial susceptibility testing. Minimum inhibitory concentrations of CAZ/AVI, MEM/VAB and IMI/REL.

Isolate n.	Minimum Inhibitory Concentration (MIC, µg/mL)		
	CAZ/AVI	MEM/VAB	IMI/REL
6/23	8/2	2/2	2/1
18/23	8/2	4/4	2/1
20/23	8/2	8/8	2/1
26/23	4/1	2/2	2/1
46/23	8/2	4/4	2/1
73/23	4/1	0.25/0.25	1/0.5
79/23	8/2	4/4	2/1
80/23	8/2	2/4	2/1
97/23	4/1	0.25/0.25	1/0.5
101/23	16/4	8/8	4/2
125/23	8/2	4/4	2/1
140/23	8/2	8/8	4/2

Abbreviations: CAZ/AVI, ceftazidime/avibactam; MEM/VAB, meropenem/vaborbactam; IMI/REL, imipenem/relebactam.

**Table 4:** Synergistic activity against planktonic *K. pneumoniae* clinical isolates. In vitro evaluation of antibiotics CAZ/AVI, MEM/VAB and IMI/REL combined with FOS, MEM, IMI or FEP was performed by checkerboard assay. The reported FICI values represent the mean of the FICIs calculated from all wells located at the turbidity/non-turbidity interface. A FICI mean ≤ 0.5 was considered synergy. Data were obtained from two independent experiments. The percentage of clinical isolates demonstrating a synergistic effect for each tested combination is also indicated.

Isolate n.	CAZ/AVI				MEM/VAB			IMI/REL		
	+FOS	+MEM	+IMI	+FEP	+FOS	+IMI	+FEP	+FOS	+MEM	+FEP
6/23	NS	S	S	S	NS	S	S	NS	NS	S
18/23	S	S	S	S	S	NS	NS	S	NS	NS
20/23	NS	S	S	S	NS	S	NS	S	NS	NS
26/23	S	S	S	S	NS	S	S	NS	NS	S
46/23	NS	S	S	S	NS	S	S	NS	NS	S
73/23	NS	S	S	S	S	S	S	S	NS	S
79/23	NS	S	S	S	NS	NS	S	NS	NS	S
80/23	NS	S	S	S	NS	NS	S	NS	NS	S
97/23	NS	S	S	S	NS	NS	S	S	S	S
101/23	NS	S	S	S	S	NS	S	NS	NS	S
125/23	NS	S	S	S	NS	S	S	NS	NS	S
140/23	NS	S	S	S	NS	NS	S	NS	NS	S
% of synergy	16.6	100	100	100	25	50	83.3	33.3	8.3	83.3

Abbreviations: CAZ/AVI, ceftazidime/avibactam; MEM/VAB, meropenem/vaborbactam; IMI/REL, imipenem/relebactam; FOS, fosfomycin; IMI, imipenem; MEM, meropenem; FEP, cefepime; FICI, fractional inhibitory concentration index; S (green), synergistic; NS (red), not synergistic.

**Table 5:** Synergistic activity against *K. pneumoniae* strong-biofilm-producing clinical isolates using checkerboard assay. In vitro evaluation of antibiotics CAZ/AVI, MEM/VAB and IMI/REL combined with FOS, MEM, IMI or FEP. FICI values for biofilm were calculated using the highest achieved BEC50/70/90 endpoints (determined via metabolic activity reduction vs. control). For off-scale endpoints (antibiotic alone), a concentration one dilution step above the maximum tested was assigned. A FICI mean ≤ 0.5 was considered synergy. Mean percentage of biofilm viability reduction from two independent experiments was used for FICI calculations. The percentage of clinical isolates demonstrating a synergistic effect for each tested combination is also indicated.

Isolate n.	CAZ/AVI				MEM/VAB			IMI/REL		
	+FOS	+MEM	+IMI	+FEP	+FOS	+IMI	+FEP	+FOS	+MEM	+FEP
6/23	NS	S	S	NS	NS	NS	NS	NS	NS	NS
18/23	NS	S	S	S	NS	S	S	NS	S	S
20/23	NS	S	S	S	NS	S	S	NS	NS	S
26/23	NS	S	S	S	NS	S	NS	NS	S	NS
46/23	NS	S	S	S	S	S	S	NS	NS	NS
73/23	NS	S	S	S	S	NS	S	NS	NS	S
101/23	NS	S	S	NS	NS	NS	S	NS	S	NS
125/23	NS	S	S	NS	NS	NS	S	NS	S	NS
% of synergy	0	100	100	62.5	25	50	75	0	50	37.5

Abbreviations: CAZ/AVI, ceftazidime/avibactam; MEM/VAB, meropenem/vaborbactam; IMI/REL, imipenem/relebactam; FOS, fosfomycin; IMI, imipenem; MEM, meropenem; FEP, cefepime; FICI, fractional inhibitory concentration index; S (green), synergistic; NS (red), not synergistic.

**Table 6:** Synergistic activity against *K. pneumoniae* strong-biofilm-producing clinical isolates using SynergyFinder 3.0 software. In vitro evaluation of antibiotics CAZ/AVI, MEM/VAB and IMI/REL combined with FOS, MEM, IMI or FEP was performed by checkerboard assay. The mean percentage of biofilm viability reduction from two independent experiments was used for synergy evaluation through the software. The highest single agent (HSA) model was applied, and combinations with synergy scores above 10 were considered synergistic (green values). Strains displaying a mean FICI > 1 in preliminary checkerboard analysis were excluded from the SynergyFinder analysis. The percentage of clinical isolates demonstrating a synergistic effect for each tested combination is also indicated.

Isolate n.	CAZ/AVI				MEM/VAB			IMI/REL		
	+FOS	+MEM	+IMI	+FEP	+FOS	+IMI	+FEP	+FOS	+MEM	+FEP
6/23	NP	29.54	27.763	19.116	NP	13.137	22.83	17.615	31.919	25.329
18/23	NP	36.077	52.382	34.023	NP	17.478	24.473	NP	37.577	37.817
20/23	NP	35.361	40.518	47.282	NP	20.844	23.126	17.929	29.794	22.339
26/23	NP	28.977	27.925	25.358	NP	17.287	17.346	NP	17.873	14.409
46/23	NP	33.876	34.023	24.082	4.504	18.817	25.028	NP	23.872	15.01
73/23	13.821	23.197	30.071	32.122	17.053	25.384	34.108	14.086	40.433	24.252
101/23	NP	22.733	23.455	21.45	NP	26.473	21.321	NP	36.069	12.942
125/23	NP	30.035	38.705	29.989	NP	13.404	24.022	6.818	22.873	13.346
% of synergy	12.5	100	100	100	12.5	100	100	37.5	100	100

Abbreviations: CAZ/AVI, ceftazidime/avibactam; MEM/VAB, meropenem/vaborbactam; IMI/REL, imipenem/relebactam; FOS, fosfomycin; IMI, imipenem; MEM, meropenem; FEP, cefepime; NP, not performed; green numbers indicate a synergistic effect; red numbers indicate a non-synergistic effect.

**Table 7:** Antibiotic concentrations at the center of the most synergistic area (MSA) against *K. pneumoniae* strong-biofilm-producing strains obtained by SynergyFinder 3.0 software analysis. MSA represents the peak synergistic landscape within a 3 × 3 dose region calculated via the software using the highest single agent (HSA) model. Comparisons among the three partner combinations for CAZ/AVI were analyzed using the Friedman test followed by Dunn’s multiple-comparison post hoc test. Pairwise comparisons for MEM/VAB and IMI/REL combinations were analyzed using the Wilcoxon matched-pairs signed-rank test. A *p*-value < 0.05 was considered statistically significant.

Isolate n.	CAZ/AVI				MEM/VAB			IMI/REL		
	+FOS	+MEM	+IMI	+FEP	+FOS	+IMI	+FEP	+FOS	+MEM	+FEP
6/23	NP	4/32	4/16	4/128	NP	1/8	2/128	2/128	2/32	4/128
18/23	NP	1/32	1/4	1/128	NP	1/16	1/128	NP	0.5/8	0.5/64
20/23	NP	1/32	1/16	2/64	NP	1/16	1/128	0.5/128	0.5/32	0.5/128
26/23	NP	4/32	4/16	4/128	NP	2/16	2/128	NP	2/32	2/128
46/23	NP	2/32	4/16	4/128	NS	2/16	2/128	NP	1/32	4/128
73/23	4/256	2/32	2/16	2/128	2/256	2/16	1/128	2/256	2/32	8/128
101/23	NP	4/32	4/16	8/128	NP	2/16	2/128	NP	2/32	8/128
125/23	NP	8/32	4/16	4/128	NP	2/16	1/128	NS	2/32	4/128
Mean MSA value ^1^	4/256	2.59/3	2.59/13.4	2.83/117.6	2/256	1.54/14.7	1.41/128	1.26/161.3	1.3/26.9	2.59/117.6

^1^ Mean values represent the geometric mean of the specific antibiotic concentrations extracted from the center of the most synergistic area (MSA) for each of the eight clinical isolates. Abbreviations: CAZ/AVI, ceftazidime/avibactam; MEM/VAB, meropenem/vaborbactam; IMI/REL, imipenem/relebactam; FOS, fosfomycin; IMI, imipenem; MEM, meropenem; FEP, cefepime; NS, not synergic; NP, not performed.

## Data Availability

The original contributions presented in this study are included in the article/Supplementary Material. Further inquiries can be directed to the corresponding authors.

## Supplementary Materials

The following supporting information can be downloaded at: https://www.mdpi.com/article/10.3390/microorganisms14081790/s1, Figure S1. Core genome phylogeny and sequence types of *K. pneumoniae* isolates. The phylogenetic tree on the left is based on the pangenome, showing the evolutionary relationships among isolates. Sequence types (STs) are indicated alongside the tree. On the right, the pangenome matrix is shown, where each column represents a gene. Blue columns indicate genes present in the isolates: core genes are shared by all samples, while accessory genes are variably present across isolates; Figure S2. Effect of CAZ/AVI treatment on strong biofilm-producing *K. pneumoniae* clinical isolates (A) 6/23, 18/23, 20/23, 26/23 and (B) 46/23, 73/23, 101/23, 125/23. Mature biofilms were treated with different concentrations of CAZ/AVI and metabolic activity (red bars) and biofilm biomass (blue bars) were evaluated through XTT reduction assay and crystal violet staining, respectively. Antibiotic concentrations exceeding the C_max_ are outlined in red. Data are expressed as mean ± SD of 3 independent experiments with 3 technical replicates; Figure S3. Effect of MEM/VAB treatment on strong biofilm-producing *K. pneumoniae* clinical isolates (A) 6/23, 18/23, 20/23, 26/23 and (B) 46/23, 73/23, 101/23, 125/23. Mature biofilms were treated with different concentrations of MEM/VAB and metabolic activity (red bars) and biofilm biomass (blue bars) were evaluated through XTT reduction assay and crystal violet staining, respectively. Antibiotic concentrations exceeding the C_max_ are outlined in red. Data are expressed as mean ± SD of 3 independent experiments with 3 technical replicates; Figure S4. Effect of IMI/REL treatment on strong biofilm-producing *K. pneumoniae* clinical isolates (A) 6/23, 18/23, 20/23, 26/23 and (B) 46/23, 73/23, 101/23, 125/23. Mature biofilms were treated with different concentrations of IMI/REL and metabolic activity (red bars) and biofilm biomass (blue bars) were evaluated through XTT reduction assay and crystal violet staining, respectively. Antibiotic concentrations exceeding the C_max_ are outlined in red. Data are expressed as mean ± SD of 3 independent experiments with 3 technical replicates; Figure S5. Four-parameter dose–response curve of CAZ/AVI. *K. pneumoniae* mature biofilms of strong biofilm-producing clinical isolates were treated with different concentrations of CAZ/AVI and biofilm biomass (blue lines) and metabolic activity (red lines) were evaluated through crystal violet (CV) staining and XTT reduction assay, respectively. Data are expressed as mean ± SD of 3 independent experiments with 3 technical replicates; Figure S6. Four-parameter dose–response curve of MEM/VAB. *K. pneumoniae* mature biofilms of strong biofilm-producing clinical isolates were treated with different concentrations of MEM/VAB and biofilm biomass (blue lines) and metabolic activity (red lines) were evaluated through crystal violet staining (CV) and XTT reduction assay, respectively. Data are expressed as mean ± SD of 3 independent experiments with 3 technical replicates; Figure S7. Four-parameter dose–response curve of IMI/REL. *K. pneumoniae* mature biofilms of strong biofilm-producing clinical isolates were treated with different concentrations of IMI/REL and biofilm bi-omass (blue lines) and metabolic activity (red lines) were evaluated through crystal violet (CV) staining and XTT reduction assay, respectively. Data are expressed as mean ± SD of 3 independent experiments with 3 technical replicates; Figure S8. Synergistic activity against *K. pneumoniae* strong biofilm-producing clinical isolates using SynergyFinder 3.0 software. In vitro evaluation of antibiotics CAZ/AVI, MEM/VAB and IMI/REL combined with FOS, MEM, IMI or FEP was performed by checkerboard assay. The mean percentage of biofilm viability reduction from 2 independent experiments was used for synergy evaluation through the software. The Highest Single Agent (HSA) model was applied, and combinations with synergy scores above 10 were considered synergistic. 2D synergy landscape map calculated using the ZIP reference model are shown. The color scale represents the synergy score, where positive values (red) indicate synergy and negative values (green/blue) indicate antagonism. The overall synergy score is indicated in the panel. The dashed black box highlights the Most Synergistic Area (MSA), indicating the optimal concentration window of both antibiotics for maximum synergistic effect; Table S1: Reads generated per samples and reads classified; Table S2: Anti-microbial resistance genes detected in *Klebsiella pneumoniae* isolates; Table S3: Plasmids, mutations and predicted mobility detected in *Klebsiella pneumoniae* isolates; Table S4: Number of total and unique genes in each sample; Table S5: Virulence factor genes detected in *Klebsiella pneumoniae* isolates; Table S6. Comparison of clinical parameters and isolate microbiological features between surviving and deceased patients (*N* = 12). Categorical data are presented as counts and percentages, while continuous data are shown as geometric means (for planktonic MICs) or arithmetic means ± standard deviation (BEC90 values). Missing MIC values were excluded from the geometric mean calculations. Biofilm eradication measurements were performed only on strong biofilm-producing strains (*n* = 5 in the survived group and *n* = 2 in the deceased group), with ambiguous non-linear regression curves/values excluded from the analysis. All planktonic MIC geometric means were calculated after log_2_ transformation and subsequent back-transformation. Statistically significant differences (*p* < 0.05) are highlighted where applicable.

## References

[1] European Centre for Disease Prevention and Control (2024). Antimicrobial Resistance in the EU/EEA (EARS-Net)-Annual Epidemiological Report 2023.

[2] Guerra M.E.S., Destro G., Vieira B., Lima A.S., Ferraz L.F.C., Hakansson A.P., Darrieux M., Converso T.R. (2022). *Klebsiella pneumoniae* Biofilms and Their Role in Disease Pathogenesis. Front. Cell. Infect. Microbiol..

[3] European Centre for Disease Prevention and Control (2024). Point Prevalence Survey of Healthcare-Associated Infections and Antimicrobial Use in European Acute Care Hospitals.

[4] European Centre for Disease Prevention and Control Healthcare-Associated Infections. https://www.ecdc.europa.eu/en/healthcare-associated-infections.

[5] Antonelli A., Giani T., Di Pilato V., Riccobono E., Perriello G., Mencacci A., Rossolini G.M. (2019). KPC-31 expressed in a ceftazidime/avibactam-resistant *Klebsiella pneumoniae* is associated with relevant detection issues. J. Antimicrob. Chemother..

[6] Hobson C.A., Pierrat G., Tenaillon O., Bonacorsi S., Bercot B., Jaouen E., Jacquier H., Birgy A. (2022). *Klebsiella pneumoniae* Carbapenemase Variants Resistant to Ceftazidime-Avibactam: An Evolutionary Overview. Antimicrob. Agents Chemother..

[7] Di Bella S., Giacobbe D.R., Maraolo A.E., Viaggi V., Luzzati R., Bassetti M., Luzzaro F., Principe L. (2021). Resistance to ceftazidime/avibactam in infections and colonisations by KPC-producing Enterobacterales: A systematic review of observational clinical studies. J. Glob. Antimicrob. Resist..

[8] Findlay J., Poirel L., Juhas M., Nordmann P. (2021). KPC-Mediated Resistance to Ceftazidime-Avibactam and Collateral Effects in *Klebsiella pneumoniae*. Antimicrob. Agents Chemother..

[9] Findlay J., Poirel L., Nordmann P. (2023). In vitro-obtained meropenem-vaborbactam resistance mechanisms among clinical *Klebsiella pneumoniae* carbapenemase-producing *K. pneumoniae* isolates. J. Glob. Antimicrob. Resist..

[10] Gaibani P., Lombardo D., Bussini L., Bovo F., Munari B., Giannella M., Bartoletti M., Viale P., Lazzarotto T., Ambretti S. (2021). Epidemiology of Meropenem/Vaborbactam Resistance in KPC-Producing *Klebsiella pneumoniae* Causing Bloodstream Infections in Northern Italy, 2018. Antibiotics.

[11] Gato E., Guijarro-Sánchez P., Alonso-García I., Pedraza-Merino R., Conde A., Lence E., Rumbo-Feal S., Peña-Escolano A., Lasarte-Monterrubio C., Blanco-Martín T. (2023). In vitro development of imipenem/relebactam resistance in KPC-producing *Klebsiella pneumoniae* involves multiple mutations including OmpK36 disruption and KPC modification. Int. J. Antimicrob. Agents.

[12] Li J., Liu Z., Wang H., Xia Y., Hu Y., Wang H., Xia F., Zou M. (2026). Ceftazidime–Avibactam Drives Mutations, Insertion, or Overexpression in *blaKPC-2* and Enhances Biofilm-Forming Ability of KPC-Producing *Klebsiella Pneumoniae*. Sci. Rep..

[13] Xie X., Chen J., Qi L., Yuan Y., Long J., Wu X., Liu Y., Guo J., Wang C., Meng X. (2025). Global Epidemiology and Resistance-Related Mutations of Ceftazidime-Avibactam-Resistant *Klebsiella Pneumoniae* Strains. Front. Cell. Infect. Microbiol..

[14] Sivori F., Francalancia M., Truglio M., Cavallo I., Pronesti C., Fabrizio G., Celesti I., Cazzani A., Furzi L., Pimpinelli F. (2025). Meropenem/vaborbactam activity against carbapenem-resistant *Klebsiella pneumoniae* from catheter-related bloodstream infections. Front. Cell. Infect. Microbiol..

[15] Birteksoz Tan A.S., Hacioglu M., Oyardi O., Olcay F.N., Inan N. (2026). In Vitro Antimicrobial Activities of Carbapenem-beta Lactamase Inhibitor Combinations Against Carbapenem-resistant Gram-negative Pathogens Isolated from Türkiye. Curr. Microbiol..

[16] Papalini C., Sabbatini S., Monari C., Mencacci A., Francisci D., Perito S., Pasticci M.B. (2020). In vitro antibacterial activity of ceftazidime/avibactam in combination against planktonic and biofilm carbapenemase-producing *Klebsiella pneumoniae* isolated from blood. J. Glob. Antimicrob. Resist..

[17] The European Committee on Antimicrobial Susceptibility Testing (2023). Routine and Extended Internal Quality Control for MIC Determination and Disk Diffusion as Recommended by EUCAST.

[18] Kim Y.C., Choi H., Kim Y.A., Park Y.S., Seo Y.H., Lee H., Lee K. (2023). Risk factors and microbiological features of recurrent *Escherichia coli* bloodstream infections. PLoS ONE.

[19] Center for Genomic Epidemiology https://www.genomicepidemiology.org/services/.

[20] BaseSpace Sequence Hub https://www.illumina.com/products/by-type/informatics-products/dragen-secondary-analysis/pipelines-apps.html.

[21] Bankevich A., Nurk S., Antipov D., Gurevich A.A., Dvorkin M., Kulikov A.S., Lesin V.M., Nikolenko S.I., Pham S., Prjibelski A.D. (2012). SPAdes: A New Genome Assembly Algorithm and Its Applications to Single-Cell Sequencing. J. Comput. Biol..

[22] Seemann T. (2014). Prokka: Rapid prokaryotic genome annotation. Bioinformatics.

[23] Letunic I., Bork P. (2024). Interactive Tree of Life (iTOL) v6: Recent Updates to the Phylogenetic Tree Display and Annotation Tool. Nucleic Acids Res..

[24] Carattoli A., Hasman H. (2020). PlasmidFinder and In Silico pMLST: Identification and Typing of Plasmid Replicons in Whole-Genome Sequencing (WGS). Horizontal Gene Transfer.

[25] Florensa A.F., Kaas R.S., Clausen P.T.L.C., Aytan-Aktug D., Aarestrup F.M. (2022). ResFinder—An open online resource for identification of antimicrobial resistance genes in next-generation sequencing data and prediction of phenotypes from genotypes. Microb. Genom..

[26] Devanga Ragupathi N.K., Muthuirulandi Sethuvel D.P., Triplicane Dwarakanathan H., Murugan D., Umashankar Y., Monk P.N., Karunakaran E., Veeraraghavan B. (2020). The Influence of Biofilms on Carbapenem Susceptibility and Patient Outcome in Device Associated *K. pneumoniae* Infections: Insights Into Phenotype vs Genome-Wide Analysis and Correlation. Front. Microbiol..

[27] European Committee on Antimicrobial Susceptibility Testing Clinical Breakpoints and Dosing of Antibiotics. https://www.eucast.org/clinical_breakpoints.

[28] Ianevski A., Giri A.K., Aittokallio T. (2022). SynergyFinder 3.0: An interactive analysis and consensus interpretation of multi-drug synergies across multiple samples. Nucleic Acids Res..

[29] Lalaoui R., Bakour S., Livnat K., Assous M.V., Diene S.M., Rolain J.-M. (2019). Spread of Carbapenem and Colistin-Resistant *Klebsiella Pneumoniae* ST512 Clinical Isolates in Israel: A Cause for Vigilance. Microb. Drug Resist..

[30] Brescini L., D’Achille G., Papalini C., Pallotta F., Teodori L., Pietrella D., Mencacci A., Canovari B., Pieretti B., Mingoia M. (2025). Clinical and molecular characteristics of KPC-producing *Klebsiella pneumoniae* bloodstream infections: Results of a multicentre study. J. Glob. Antimicrob. Resist..

[31] García-Fernández A., Villa L., Carta C., Venditti C., Giordano A., Venditti M., Mancini C., Carattoli A. (2012). *Klebsiella Pneumoniae* ST258 Producing KPC-3 Identified in Italy Carries Novel Plasmids and OmpK36/OmpK35 Porin Variants. Antimicrob. Agents Chemother..

[32] Xu Q., Xie M., Yang X., Liu X., Ye L., Chen K., Chan E.W.-C., Chen S. (2024). Conjugative Transmission of Virulence Plasmid in *Klebsiella Pneumoniae* Mediated by a Novel IncN-like Plasmid. Microbiol. Res..

[33] Li Y., Ni M. (2023). Regulation of biofilm formation in *Klebsiella pneumoniae*. Front. Microbiol..

[34] Zaborskytė G., Coelho P., Wrande M., Sandegren L. (2026). Rapid Evolution of *Klebsiella Pneumoniae* Biofilms in Vitro Delineates Adaptive Changes Selected during Infection. Nat. Commun..

[35] Villa L., Feudi C., Fortini D., Brisse S., Passet V., Bonura C., Endimiani A., Mammina C., Ocampo A.M., Jimenez J.N. (2017). Diversity, Virulence, and Antimicrobial Resistance of the KPC-Producing *Klebsiella Pneumoniae* ST307 Clone. Microb. Genom..

[36] Palomba E., Comelli A., Saluzzo F., Di Marco F., Matarazzo E., Re N.L., Bielli A., Vismara C.S., Muscatello A., Rossi M. (2025). Activity of imipenem/relebactam against KPC-producing *Klebsiella pneumoniae* and the possible role of Ompk36 mutation in determining resistance: An Italian retrospective analysis. Ann. Clin. Microbiol. Antimicrob..

[37] Fu Y., Zhang Y., Zhao J., Xie J., Kong H., Yang T., Chen W., Xu M. (2025). Emergence of Co-Resistance to Imipenem/Relebactam and Ceftazidime/Avibactam in Clinical *Klebsiella Pneumoniae* ST11 Clone Due to KPC-2 N132S and CTX-M-65 S130G/P167S Substitutions. Antimicrob. Agents Chemother..

[38] Cerdeira L.T., Cunha M.P.V., Francisco G.R., Bueno M.F.C., Araujo B.F., Ribas R.M., Gontijo-Filho P.P., Knöbl T., De Oliveira Garcia D., Lincopan N. (2017). IncX3 Plasmid Harboring a Non-Tn 4401 Genetic Element (NTE KPC) in a Hospi-tal-Associated Clone of KPC-2-Producing *Klebsiella Pneumoniae* ST340/CG258. Diagn. Microbiol. Infect. Dis..

[39] Patil S., Chen H., Guo C., Zhang X., Ren P.-G., Francisco N.M., Wen F. (2021). Emergence of *Klebsiella Pneumoniae* ST307 Co-Producing CTX-M with SHV and KPC from Paediatric Patients at Shenzhen Children’s Hospital, China. IDR.

[40] Findlay J., Rens C., Poirel L., Nordmann P. (2022). In Vitro Mechanisms of Resistance Development to Imipenem-Relebactam in KPC-Producing *Klebsiella pneumoniae*. Antimicrob. Agents Chemother..

[41] Gaibani P., Giani T., Bovo F., Lombardo D., Amadesi S., Lazzarotto T., Coppi M., Rossolini G.M., Ambretti S. (2022). Resistance to Ceftazidime/Avibactam, Meropenem/Vaborbactam and Imipenem/Relebactam in Gram-Negative MDR Bacilli: Molecular Mechanisms and Susceptibility Testing. Antibiotics.

[42] Lomovskaya O., Sun D., Rubio-Aparicio D., Nelson K., Tsivkovski R., Griffith D.C., Dudley M.N. (2017). Vaborbactam: Spectrum of Beta-Lactamase Inhibition and Impact of Resistance Mechanisms on Activity in *Enterobacteriaceae*. Antimicrob. Agents Chemother..

[43] Boattini M., Comini S., Ricciardelli G., Pastrone L., Casale R., Guarrasi L., Cavallo R., Costa C., Gaibani P., Bianco G. (2026). Emergence of Resistance to Novel β-Lactam/β-Lactamase Inhibitor Combinations in KPC-Producing *Klebsiella Pneumoniae*: Clinical and Genomic Insights from Consecutive Bloodstream Infections. Infection.

[44] Tedeschi S., Giannitsioti E., Mayer C. (2025). Emerging Concepts for the Treatment of Biofilm-Associated Bone and Joint Infections with IV Fosfomycin: A Literature Review. Microorganisms.

[45] Sutaria D.S., Moya B., Green K.B., Kim T.H., Tao X., Jiao Y., Louie A., Drusano G.L., Bulitta J.B. (2018). First Penicillin-Binding Protein Occupancy Patterns of β-Lactams and β-Lactamase Inhibitors in *Klebsiella pneumoniae*. Antimicrob. Agents Chemother..

[46] Zheng M., Li F.H., Liu J., Li W.J., Yin R.X., Cai D.T., Andrey D.O., Zheng S.L., Gales A.C., Zhang W.J. (2024). Synergistic effects of ceftazidime/avibactam combined with meropenem in a murine model of infection with KPC-producing *Klebsiella pneumoniae*. J. Antimicrob. Chemother..

[47] Kang Y., Kang S., Cui J. (2026). Combination of Ceftazidime-Avibactam and Meropenem for the Treatment of Pneumonia and Bloodstream Infections Caused by Pan-Drug Resistant *Pseudomonas aeruginosa* ST244: A Case Report. Infect. Drug Resist..

[48] Kuai J., Zhang Y., Lu B., Chen H., Zhang Y., Li H., Wang Y., Wang Q., Wang H., Wang X. (2023). In vitro Synergistic Activity of Ceftazidime-Avibactam in Combination with Aztreonam or Meropenem Against Clinical Enterobacterales Producing blaKPC or blaNDM. Infect. Drug Resist..

[49] Li Y.Y., Wang J., Wang R., Cai Y. (2020). Double-carbapenem therapy in the treatment of multidrug resistant Gram-negative bacterial infections: A systematic review and meta-analysis. BMC Infect. Dis..

[50] Oliva A., Gizzi F., Mascellino M.T., Cipolla A., D’Abramo A., D’Agostino C., Trinchieri V., Russo G., Tierno F., Iannetta M. (2016). Bactericidal and synergistic activity of double-carbapenem regimen for infections caused by carbapenemase-producing *Klebsiella pneumoniae*. Clin. Microbiol. Infect..

[51] Soanker R., Vemu L., Vimala S., Kanne P., Thumma V.M., Chavala G. (2024). The Effect of Double Carbapenem Regimen in the Management of Carbapenem-Resistant *Klebsiella pneumoniae* Infections: A Report of Five Cases. Cureus.

[52] De Pascale G., Martucci G., Montini L., Panarello G., Cutuli S.L., Di Carlo D., Di Gravio V., Di Stefano R., Capitanio G., Vallecoccia M.S. (2017). Double carbapenem as a rescue strategy for the treatment of severe carbapenemase-producing *Klebsiella pneumoniae* infections: A two-center, matched case-control study. Crit. Care.

[53] Moody R. (2021). The effect of double-carbapenem therapy on mortality rates and microbiological cure rates in patients diagnosed with carbapenem-resistant *Klebsiella pneumoniae* infections in comparison to monotherapy and currently used combinations of antibiotics. J. Med. Res. Innov..

[54] Comini S., Boattini M., Gaibani P., Bianco G. (2026). Cefepime combined with late-generation β-lactamase inhibitors: Mechanisms of action, in vitro activity, PK/PD characteristics, clinical evidence and resistance mechanisms. Antibiotics.

[55] Caiazzo L., Carretto E., Gaibani P. (2025). In vitro activity of cefepime/enmetazobactam against *Klebsiella pneumoniae* carrying *blaKPC* allelic variants conferring resistance to ceftazidime/avibactam. Int. J. Antimicrob. Agents.

[56] Dutkiewicz M., Jousset A.B., de Swardt H., Rezzoug I., A Bonnin R., Dortet L., Emeraud C. (2026). In vitro susceptibility of cefepime-enmetazobactam, cefepime-taniborbactam and cefepime-zidebactam towards carbapenemase-producing Enterobacterales. J. Antimicrob. Chemother..

[57] Bhowmick T., Canton R., Pea F., Quevedo J., Santerre Henriksen A., Timsit J.F., Kaye K.S. (2025). Cefepime-enmetazobactam: First approved cefepime-β- lactamase inhibitor combination for multi-drug resistant Enterobacterales. Future Microbiol..

[58] Crema A., Shafique A., Bianco G., Gaibani P. (2026). Synergistic activity of cefepime/enmetazobactam with meropenem, piperacillin, piperacillin/tazobactam, cefiderocol and fosfomycin against *Klebsiella pneumoniae* carrying *blaKPC* allelic variants. J. Antimicrob. Chemother..

[59] Zhang S., Yang J., Yang Q., Li Q., Zhong Z., Wang M., Jia R., Chen S., Liu M., Zhu D. (2025). High Prevalence of Plas-mid-Mediated Fosfomycin Resistance in Waterfowl-Derived *Escherichia Coli* Strains: Insights into Genetic Context and Transmission Dynamics in China. Front. Vet. Sci..

