# Supplementary material for: Targeting KPC-Producing Klebsiella pneumoniae Biofilms: Genomic Profiles, Clinical Outcomes and Novel Combinations
Source: Microorganisms. 2026 Aug 14;14(8):1790. doi: 10.3390/microorganisms14081790 (PMC13515105; doi:10.3390/microorganisms14081790)
Supplement: Supplementary file 1 [file microorganisms-14-01790-s001.zip › microorganisms-4402733-supplementary.pdf]

Table of contents

Supplementary data on *K. pneumoniae* sequencing 2
Figure S1 5
Figure S2 6
Figure S3 8
Figure S4 10
Figure S5 12
Figure S6 13
Figure S7 14
Figure S8 15
Table S1 19
Table S2 20
Table S3 22
Table S4 24
Table S5 25
Table S6 27

Supplementary data on *K. pneumoniae* sequencing

1. Results

1.1 Sequencing Output and Genome Assembly

Whole-genome sequencing of the 12 clinical isolates generated 27308185 raw reads, whose 97.17% (25457441) passed Illumina quality filtering. Quality assessment showed that 93.9% of bases achieved Q30 or higher, confirming high accuracy of data. The number of reads per isolate ranged from 1.29 million (sample 101/23) to 2.73 million (sample 18/23) (Table S1). This sequencing depth was sufficient for reliable genome assembly and downstream comparative genomic analysis. De novo assembly produced genome sizes ranging between approximately 5.1 and 5.3 Mb, consistent with the expected genome size of *K. pneumoniae*. Taxonomic assignment of sequence reads using the DRAGEN Metagenomics Pipeline confirmed that all isolates belonged to the genus *Klebsiella* and specifically to the species *K. pneumoniae*. No contamination from non-Klebsiella species was detected, confirming the integrity of the sequencing data.

1.2 Antimicrobial Resistance Determinants

Anti-microbial resistance genes detected in clinical isolates are reported in Table S2. All isolates carried *blaKPC* and the *fosA* genes, which confer resistance to carbapenems and fosfomycin, respectively. Isolates 73/23 and 80/23, similar to each other, exhibited the broadest resistance profiles, harboring additional aminoglycoside resistance genes aph(6)-Id, aac(6')-Ib-cr, aph(3'')-Ib, extended-spectrum beta-lactamase genes *blaOXA-1, blaSHV-100, blaTEM-1B, blaCTX-M-15*, as well *sul2* and *dfrA14* confering resistance to sulfonamides and trimethoprim respectively. Isolates n. 6/23, 18/23, 20/23, 26/23, 46/23, 101/23, and 125/23 carried *dfrA12*, indicating trimethoprim resistance. Efflux pump genes (OqxAB) were detected in all isolates except 73/23, providing additional multidrug resistance to fluoroquinolones and quinolones. Mutational analysis of porin genes revealed several substitutions in *ompK36* and *ompK37*. In *ompK36* gene two mutation have been detected: A217S (isolates 6/23, 79/23, 101/23, 140/23) and N218H (isolates 79/23 and 140/23). All isolates had I70M and I128M substitutions in *ompK37* gene and in some cases we identified additional mutations: N230G (isolates 6/23, 73/23, 80/23, 125/23), T234H (isolates 6/23, 18/23, 80/23, 101/23, 125/23), Q235Y (isolates 79/23, 125/23).

1.3 Plasmid Content and Distribution

Plasmid analysis revealed extensive plasmid diversity. Plasmids belonging to the incompatibility (Inc) group B (IncFIB) were present in all the isolates (Table S3). IncFII(K) plasmids were found in isolates n. 6/23, 18/23, 26/23, 46/23, 73/23, 80/23, 87/23, 101/23, 125/23, while IncX3 plasmids were detected in 6/23, 18/23, 20/23, 26/23, 46/23, 101/23, 125/23.

Some plasmids were isolate-specific. Col156 plasmid (member of the ColE-type family of small plasmids) and IncHI1B(pNDM-MAR) (belongs to the IncHI1B incompatibility group) were detected only in isolate 79/23; Col440II (another member of the ColE-type small plasmid family) was detected only in isolate 140/23; and ColRNAI (another member of the ColE-type small plasmid family) was detected in isolates 79/23 and 140/23.

1.4 Population Structure and Comparative Genomics

Multi-locus sequence typing (MLST), performed on seven specific genes for *K. pneumoniae* (*gapA, infB, mdh, pgi, phoE, rpoB, tonB*) classified the isolates into three major sequence types: ST512 (6/23, 18/23, 20/23, 26/23, 46/23, 101/23, 125/23), ST307 (73/23, 80/23, 97/23), and ST101 (79/23, 140/23) (Figure S1).

Pan-genome analysis identified 6942 genes across all the isolates, whose 4243 (61%) represented the core genome. The number of unique genes varied considerably between isolates from 199 (strain 80/23) and 349 genes (strain 79/23) (Table S4). Phylogenetic analysis based on core genome alignments showed clustering of isolates according to their sequence type. The ST512 strains formed a highly conserved cluster, consistent with clonal dissemination. The ST307 cluster was genetically distinct, highlighting its emergence as a high-risk international clone. The ST101 isolates displayed the greatest accessory genome diversity, reflecting their potential for adaptive evolution.

1.5 Virulence Determinants and Biofilm-Associated Genes

Screening for virulence factors involved in biofilm formation revealed the presence of conserved genes across all the isolates (Table S5): *galF* (capsule production), *rpmA* (mucoid phenotype regulation), *fimA-H*, *mrkD* (fimbrial adhesins), and *acrAB* (efflux-mediated phagocytosis inhibition). All the bacterial isolates carried *iutA*, the aerobactin receptor gene, while the complete aerobactin operon (iucA-D) was found only in isolate n. 79/23.

Genes associated with biofilm formation were widely distributed. The *wcaJ* gene, involved in colanic acid synthesis and biofilm development, was present only in isolates 79/23 and 140/23.

2. References

[1] Center for Genomic Epidemiology. https://www.genomicepidemiology.org/services/ [accessed 11 August 2024].

[2] BaseSpace Sequence Hub. https://basespace.illumina.com/apps/16640624/DRAGEN-Metagenomics-Pipeline?preferredversion= [accessed 29 August 2025]

[3] Bankevich A, Nurk S, Antipov D, Gurevich AA, Dvorkin M, Kulikov AS, et al. SPAdes: A New Genome Assembly Algorithm and Its Applications to Single-Cell Sequencing. J Comput Biol. 2012;19(5):455-477. https://doi.org/10.1089/cmb.2012.0021

[4] Seemann T. Prokka: rapid prokaryotic genome annotation. Bioinformatics. 2014;30(14):2068-2069. DOI:https://doi.org/10.1093/bioinformatics/btu153

[5] Carattoli A, Hasman H. PlasmidFinder and In Silico pMLST: Identification and Typing of Plasmid Replicons in Whole-Genome Sequencing (WGS). Methods Mol Biol Clifton NJ. 2020;2075:285-294. DOI:https://doi.org/10.1007/978-1-4939-9877-7_20

[6] Florensa AF, Kaas RS, Clausen PTLC, Aytan-Aktug D, Aarestrup FM. ResFinder – an open online resource for identification of antimicrobial resistance genes in next-generation sequencing data and prediction of phenotypes from genotypes. Microb Genomics. 2022;8(1):000748. https://doi.org/10.1099/mgen.0.000748

Figure S1. Core genome phylogeny and sequence types of *K. pneumoniae* isolates. The phylogenetic tree on the left is based on the pangenome, showing the evolutionary relationships among isolates. Sequence types (STs) are indicated alongside the tree. On the right, the pangenome matrix is shown, where each column represents a gene. Blue columns indicate genes present in the isolates: core genes are shared by all samples, while accessory genes are variably present across isolates.

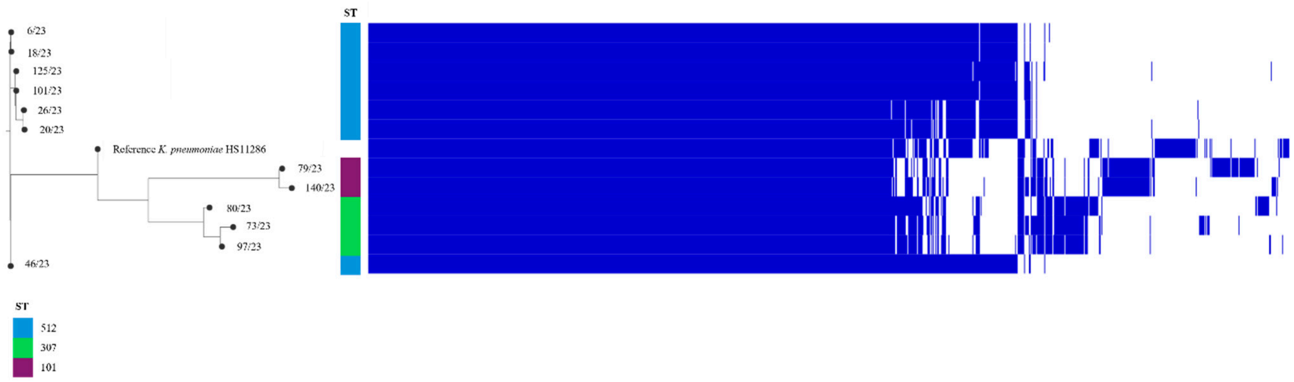

Figure S2. Effect of CAZ/AVI treatment on strong biofilm-producing *K. pneumoniae* clinical isolates (A) 6/23, 18/23, 20/23, 26/23 and (B) 46/23, 73/23, 101/23, 125/23. Mature biofilms were treated with different concentrations of CAZ/AVI and metabolic activity (red bars) and biofilm biomass (blue bars) were evaluated through XTT reduction assay and crystal violet staining, respectively. Antibiotic concentrations exceeding the C_{max} are outlined in red. Data are expressed as mean ±SD of 3 independent experiments with 3 technical replicates.

A

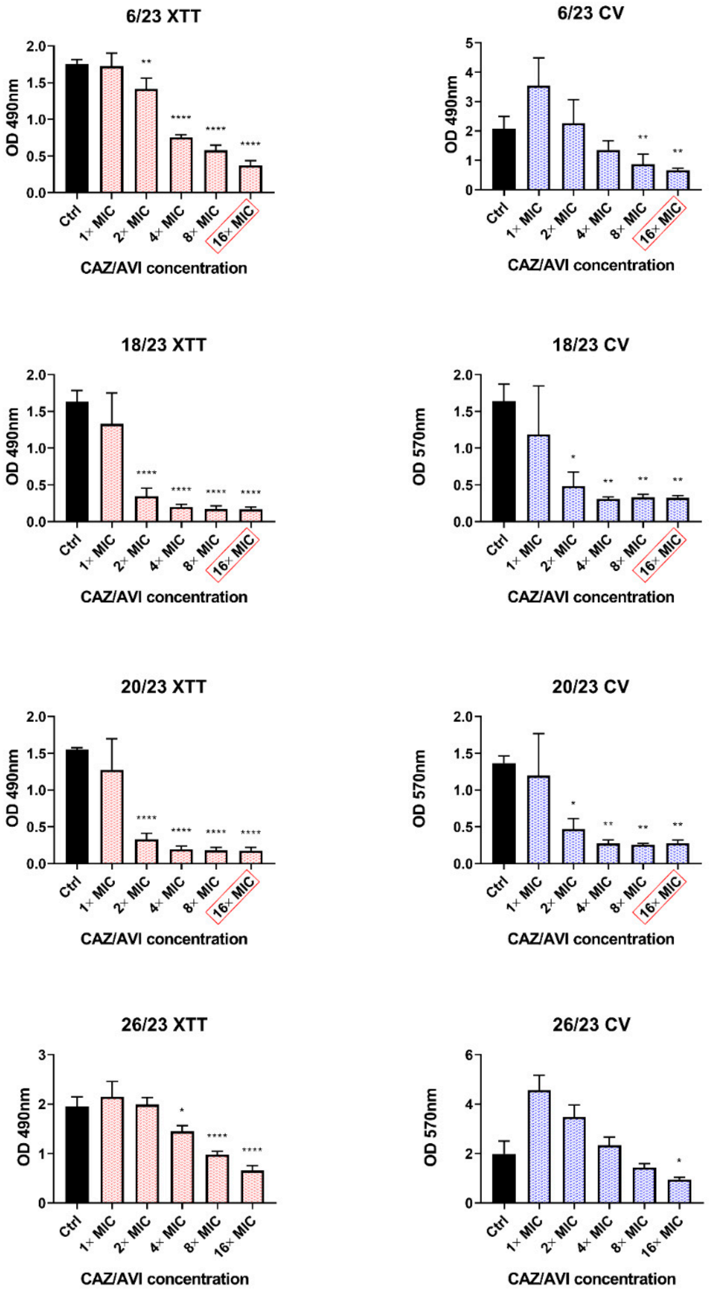

B

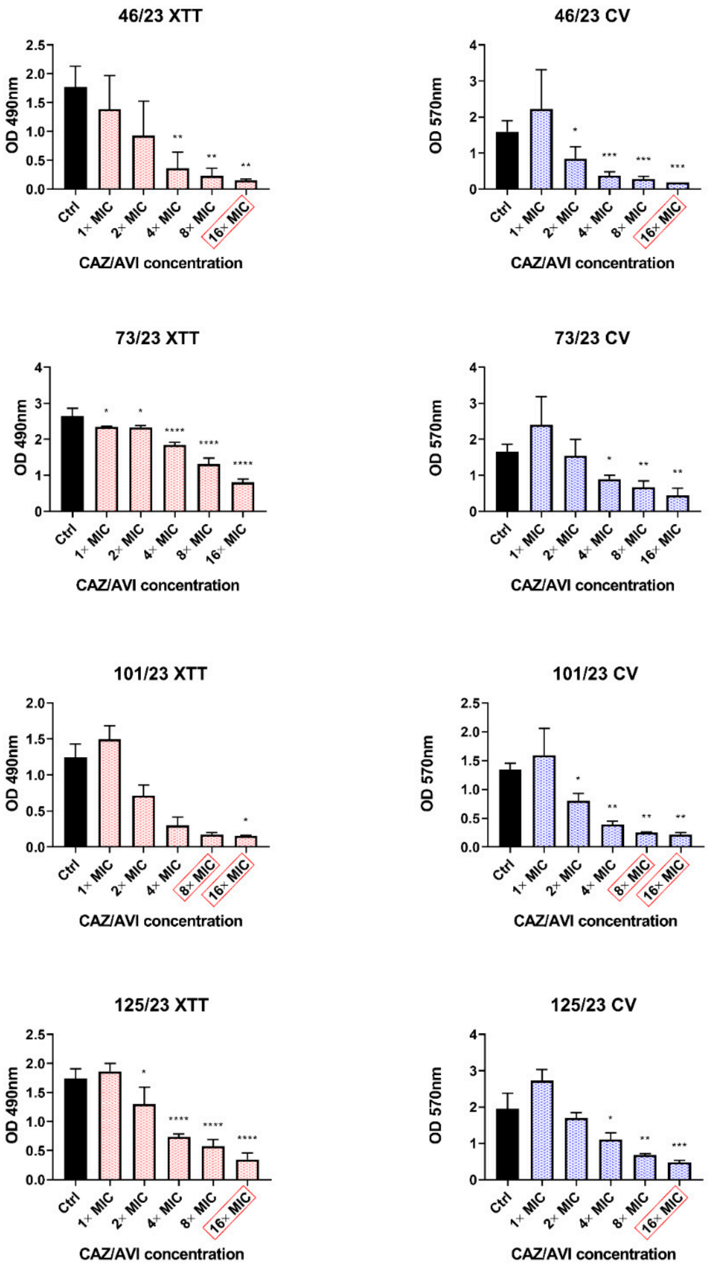
46/23 XTT
OD 490nm
CAZ/AVI concentration
46/23 CV
OD 570nm
CAZ/AVI concentration
73/23 XTT
OD 490nm
CAZ/AVI concentration
73/23 CV
OD 570nm
CAZ/AVI concentration
101/23 XTT
OD 490nm
CAZ/AVI concentration
101/23 CV
OD 570nm
CAZ/AVI concentration
125/23 XTT
OD 490nm
CAZ/AVI concentration
125/23 CV
OD 570nm
CAZ/AVI concentration
Ctrl
1× MIC
2× MIC
4× MIC
8× MIC
16× MIC

Figure S3. Effect of MEM/VAB treatment on strong biofilm-producing *K. pneumoniae* clinical isolates (A) 6/23, 18/23, 20/23, 26/23 and (B) 46/23, 73/23, 101/23, 125/23. Mature biofilms were treated with different concentrations of MEM/VAB and metabolic activity (red bars) and biofilm biomass (blue bars) were evaluated through XTT reduction assay and crystal violet staining, respectively. Antibiotic concentrations exceeding the C_{max} are outlined in red. Data are expressed as mean ±SD of 3 independent experiments with 3 technical replicates.

A

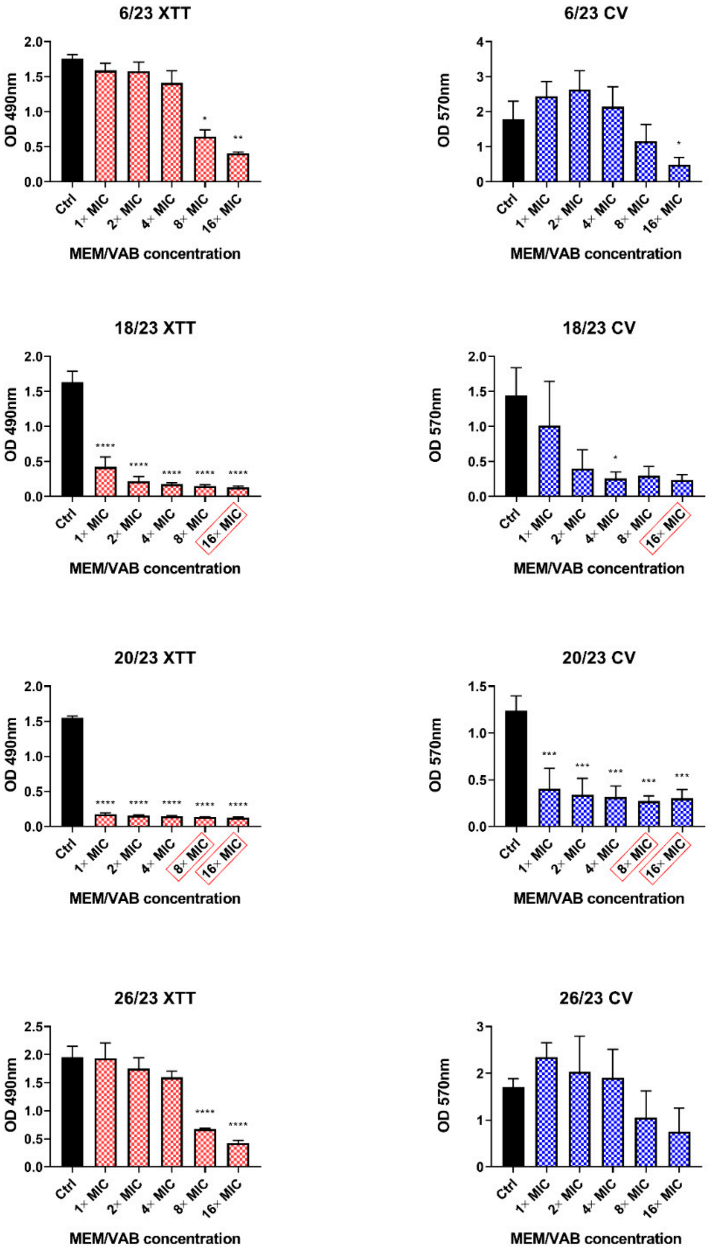

B

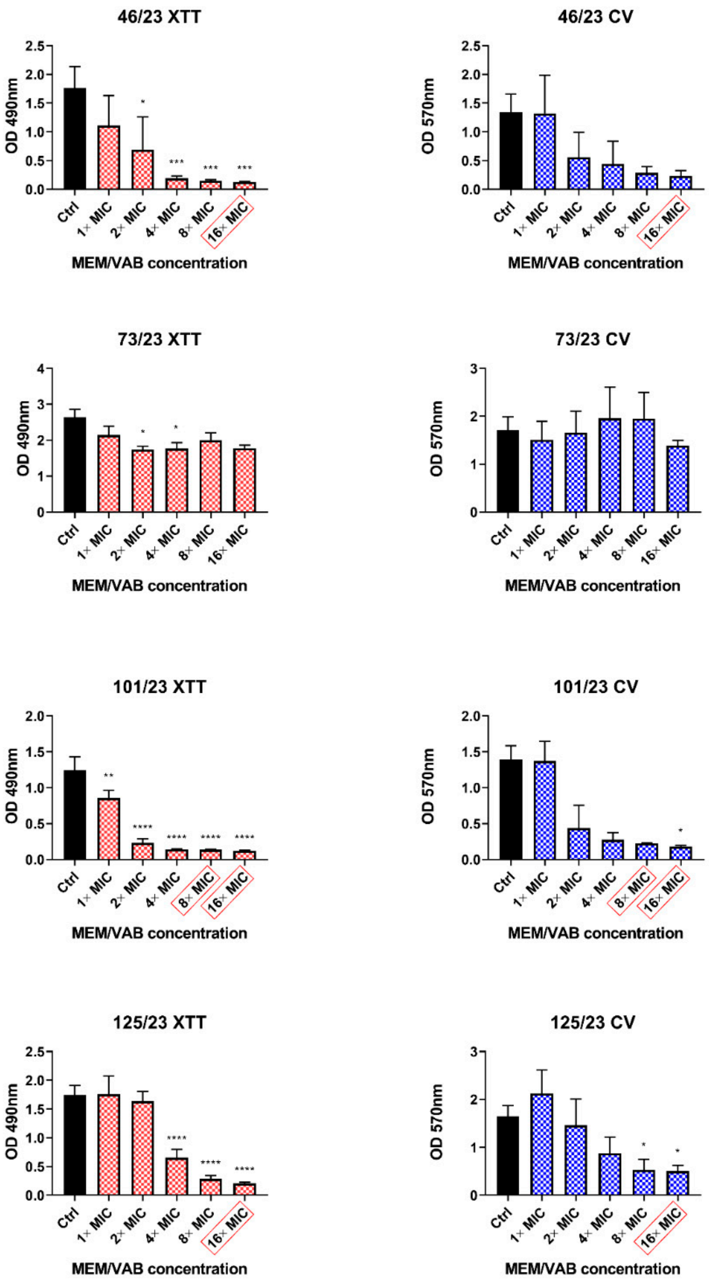
46/23 XTT
OD 490nm
MEM/VAB concentration
46/23 CV
OD 570nm
MEM/VAB concentration
73/23 XTT
OD 490nm
MEM/VAB concentration
73/23 CV
OD 570nm
MEM/VAB concentration
101/23 XTT
OD 490nm
MEM/VAB concentration
101/23 CV
OD 570nm
MEM/VAB concentration
125/23 XTT
OD 490nm
MEM/VAB concentration
125/23 CV
OD 570nm
MEM/VAB concentration
Ctrl
1× MIC
2× MIC
4× MIC
8× MIC
16× MIC

Figure S4. Effect of IMI/REL treatment on strong biofilm-producing *K. pneumoniae* clinical isolates (A) 6/23, 18/23, 20/23, 26/23 and (B) 46/23, 73/23, 101/23, 125/23. Mature biofilms were treated with different concentrations of IMI/REL and metabolic activity (red bars) and biofilm biomass (blue bars) were evaluated through XTT reduction assay and crystal violet staining, respectively. Antibiotic concentrations exceeding the C_{max} are outlined in red. Data are expressed as mean ±SD of 3 independent experiments with 3 technical replicates.

A

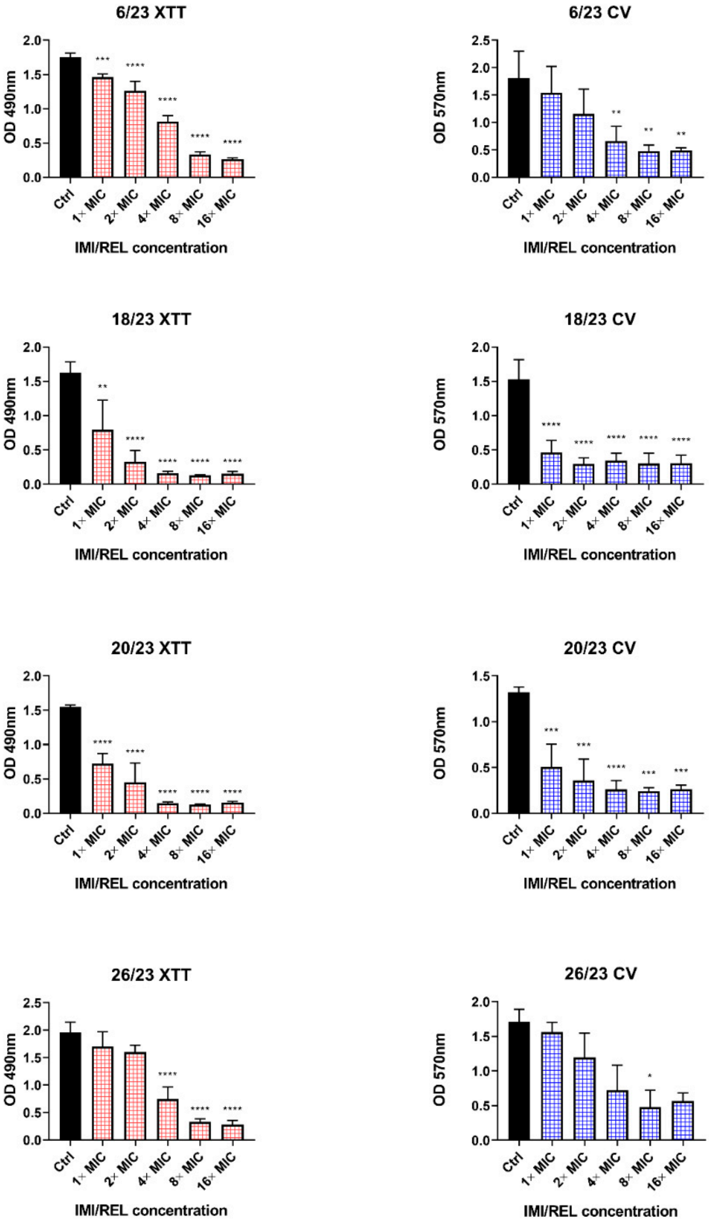

B

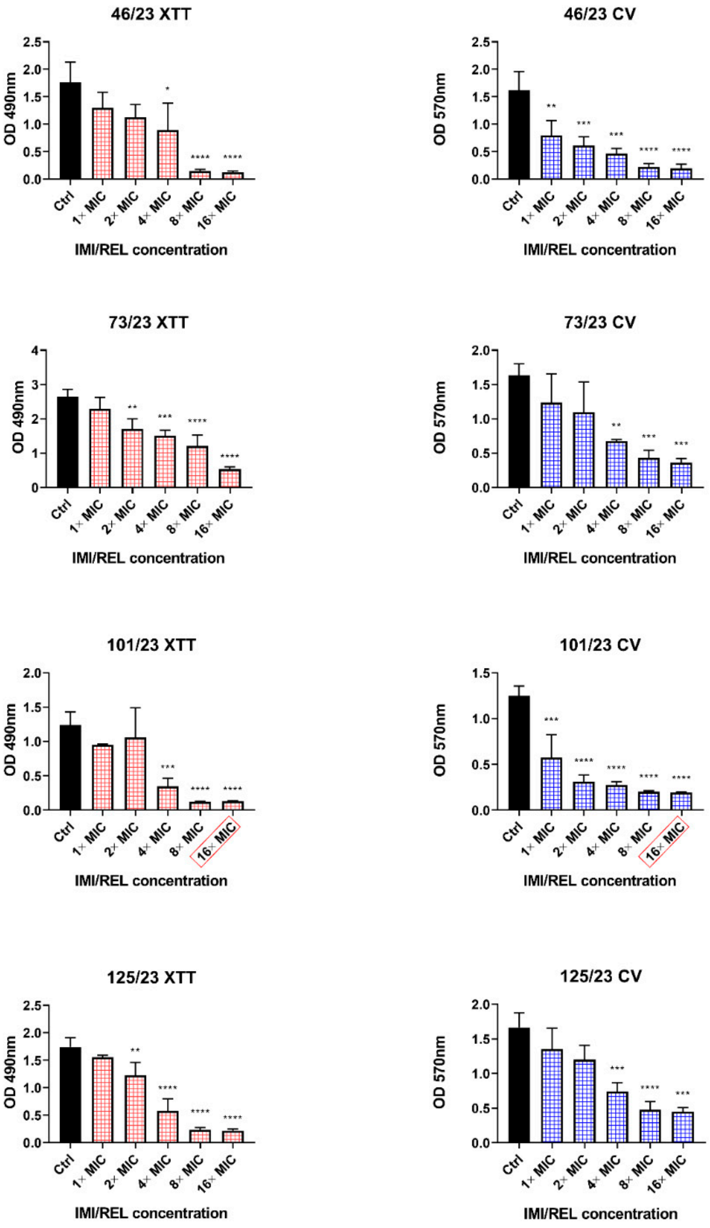

46/23 XTT
46/23 CV
73/23 XTT
73/23 CV
101/23 XTT
101/23 CV
125/23 XTT
125/23 CV
OD 490nm
OD 570nm
Ctrl
1× MIC
2× MIC
4× MIC
8× MIC
16× MIC
IMI/REL concentration

Figure S5. Four-parameter dose–response curve of CAZ/AVI. *K. pneumoniae* mature biofilms of strong biofilm-producing clinical isolates were treated with different concentrations of CAZ/AVI and biofilm biomass (blue lines) and metabolic activity (red lines) were evaluated through crystal violet (CV) staining and XTT reduction assay, respectively. Data are expressed as mean ± SD of 3 independent experiments with 3 technical replicates.

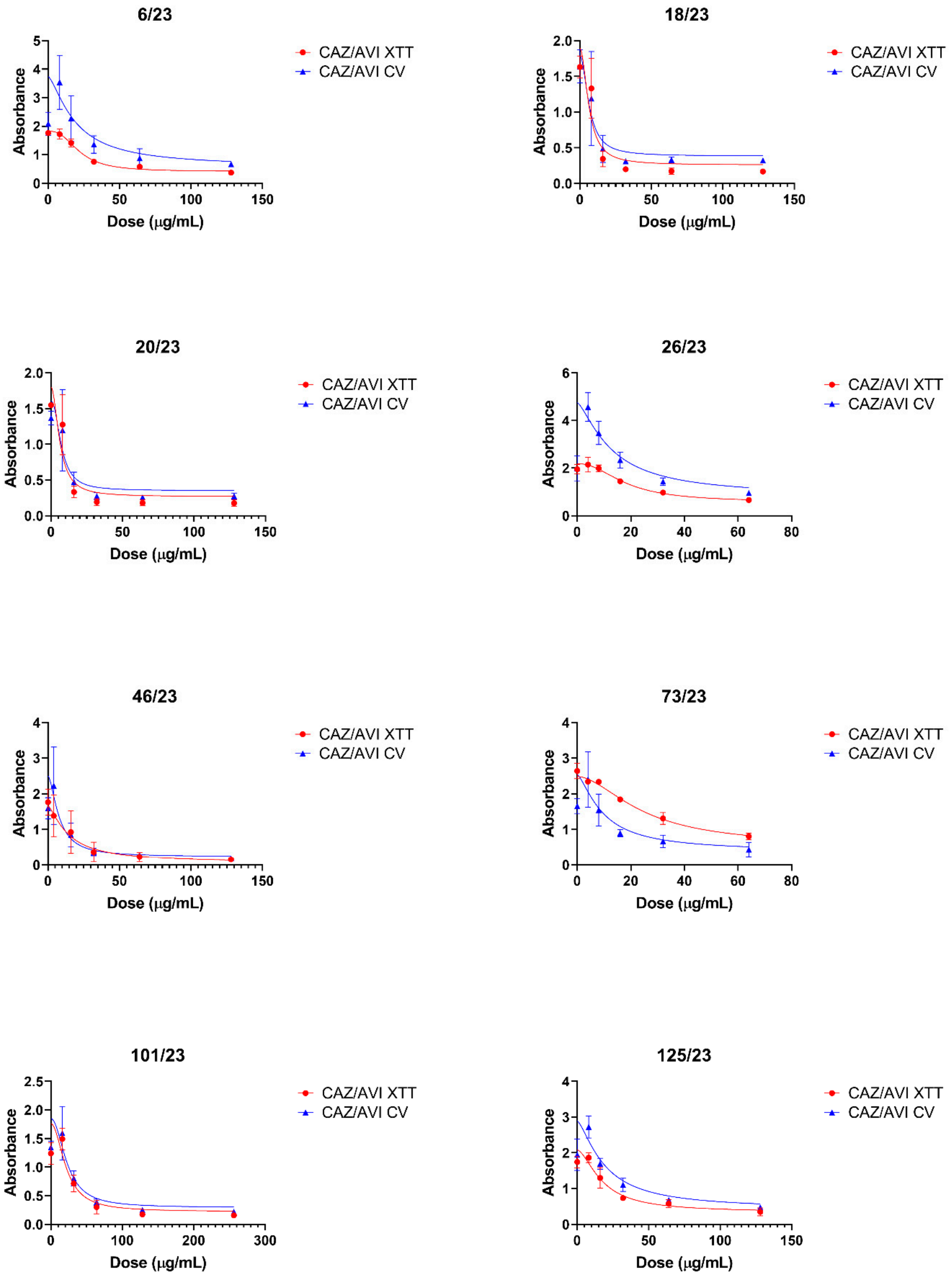

Figure S6. Four-parameter dose–response curve of MEM/VAB. *K. pneumoniae* mature biofilms of strong biofilm-producing clinical isolates were treated with different concentrations of MEM/VAB and biofilm biomass (blue lines) and metabolic activity (red lines) were evaluated through crystal violet staining (CV) and XTT reduction assay, respectively. Data are expressed as mean ±SD of 3 independent experiments with 3 technical replicates.

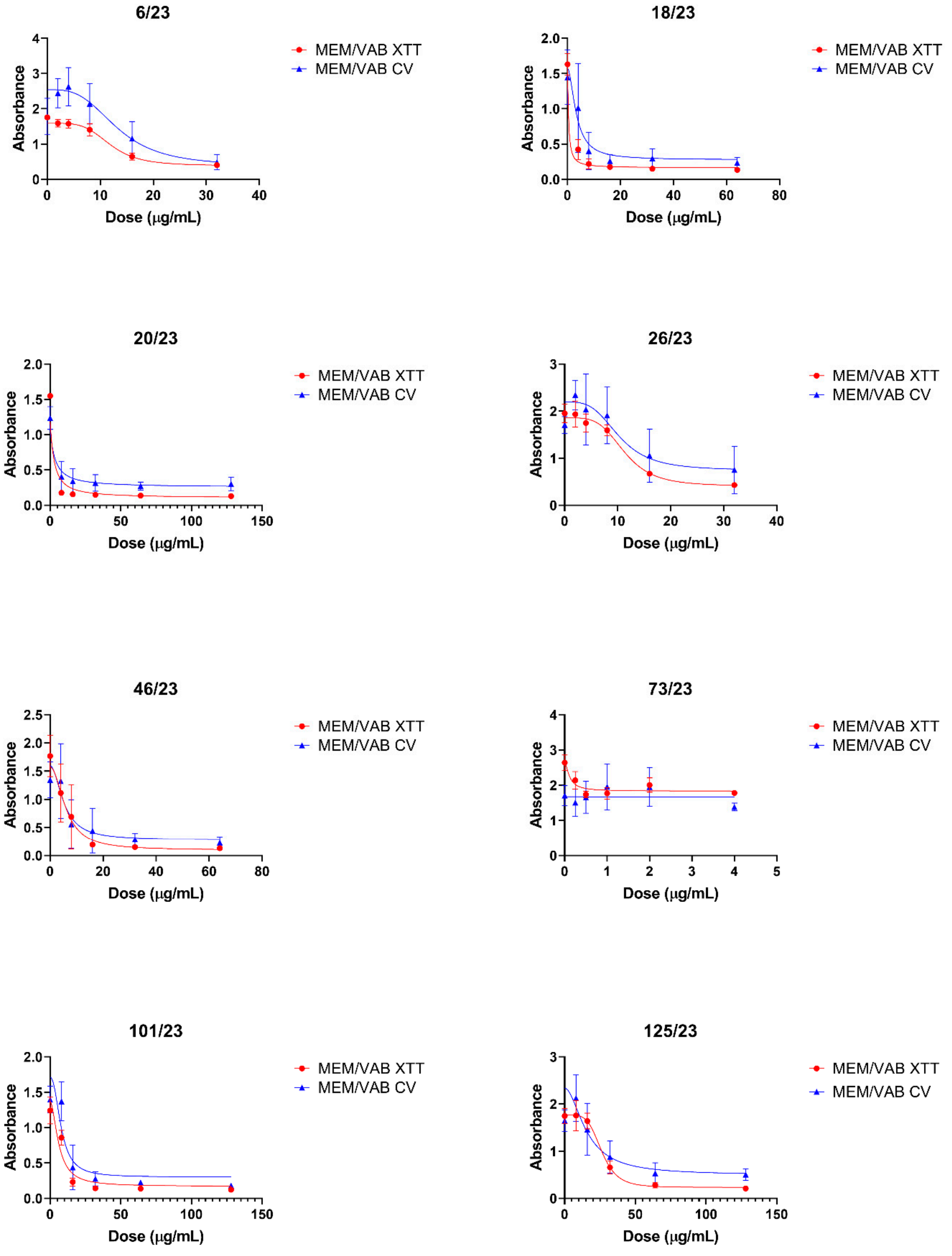

Figure S7. Four-parameter dose–response curve of IMI/REL. *K. pneumoniae* mature biofilms of strong biofilm-producing clinical isolates were treated with different concentrations of IMI/REL and biofilm bi-omass (blue lines) and metabolic activity (red lines) were evaluated through crystal violet (CV) staining and XTT reduction assay, respectively. Data are expressed as mean ±SD of 3 independent experiments with 3 technical replicates.

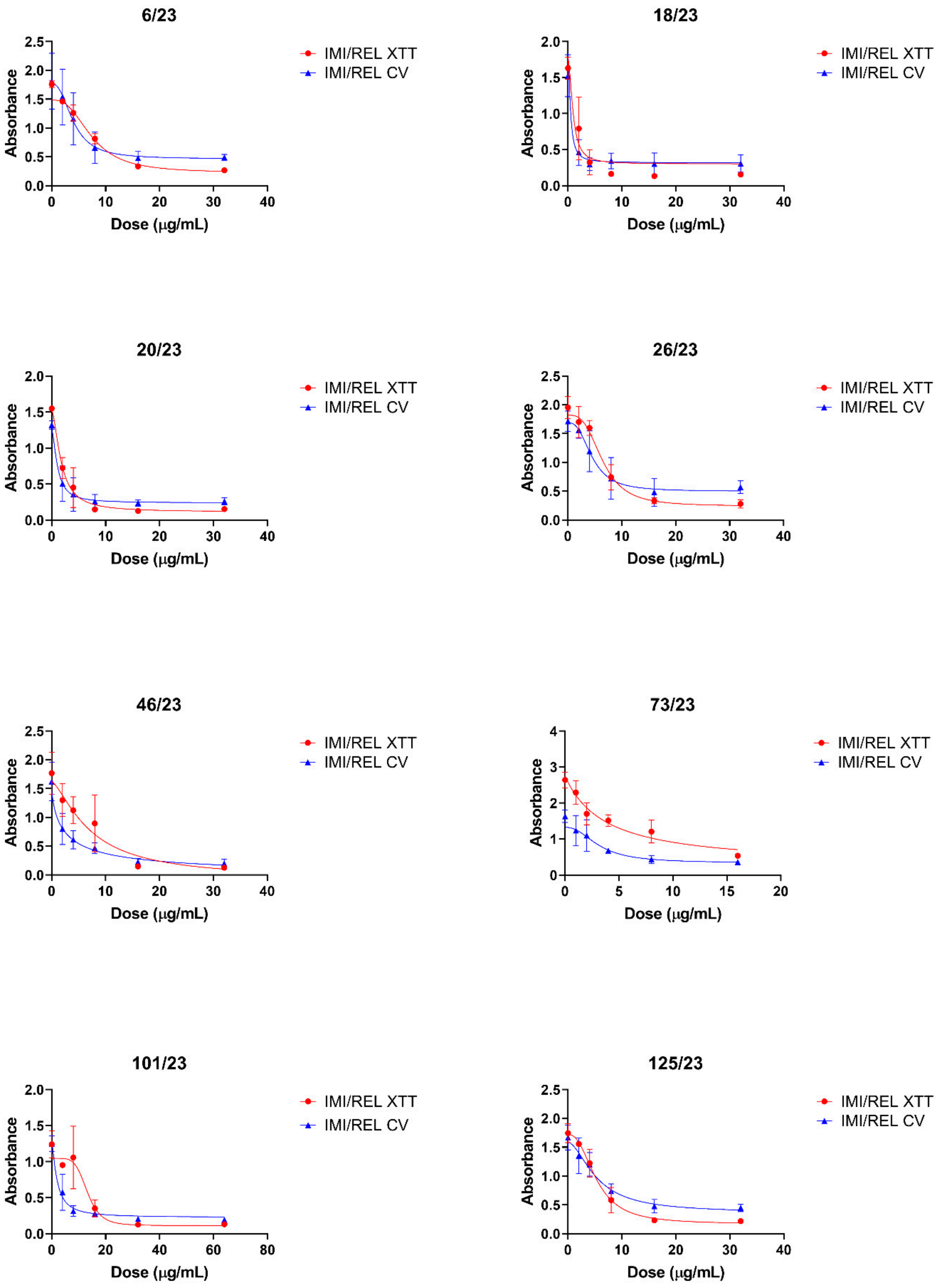

Figure S8. Synergistic activity against *K. pneumoniae* strong biofilm-producing clinical isolates using SynergyFinder 3.0 software. In vitro evaluation of antibiotics CAZ/AVI, MEM/VAB and IMI/REL combined with FOS, MEM, IMI or FEP was performed by checkerboard assay. The mean percentage of biofilm viability reduction from 2 independent experiments was used for synergy evaluation through the software. The Highest Single Agent (HSA) model was applied, and combinations with synergy scores above 10 were considered synergistic. 2D synergy landscape map calculated using the ZIP reference model are shown. The color scale represents the synergy score, where positive values (red) indicate synergy and negative values (green/blue) indicate antagonism. The overall synergy score is indicated in the panel. The dashed black box highlights the Most Synergistic Area (MSA), indicating the optimal concentration window of both antibiotics for maximum synergistic effect.

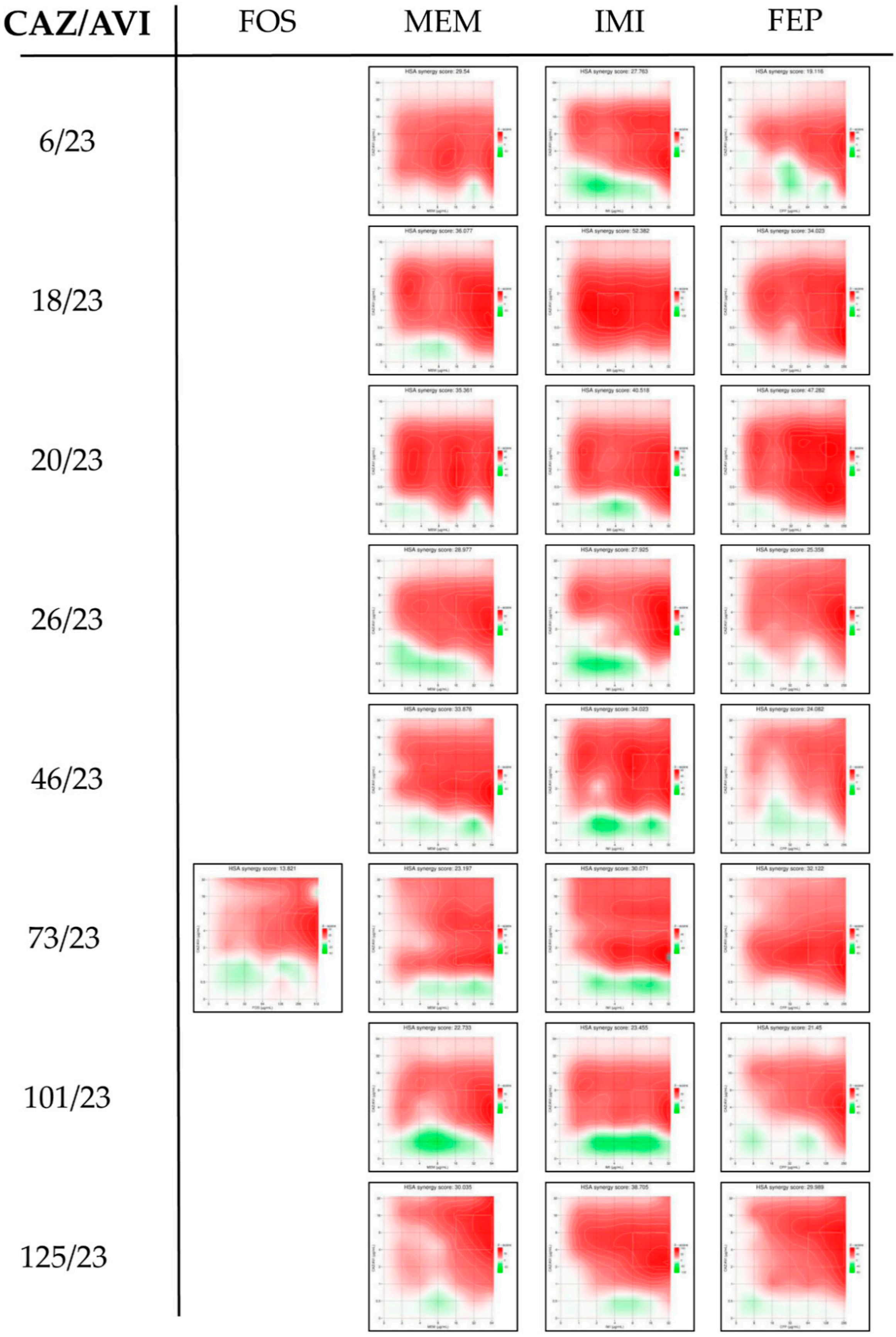
CAZ/AVI
FOS
MEM
IMI
FEP
6/23
18/23
20/23
26/23
46/23
73/23
101/23
125/23

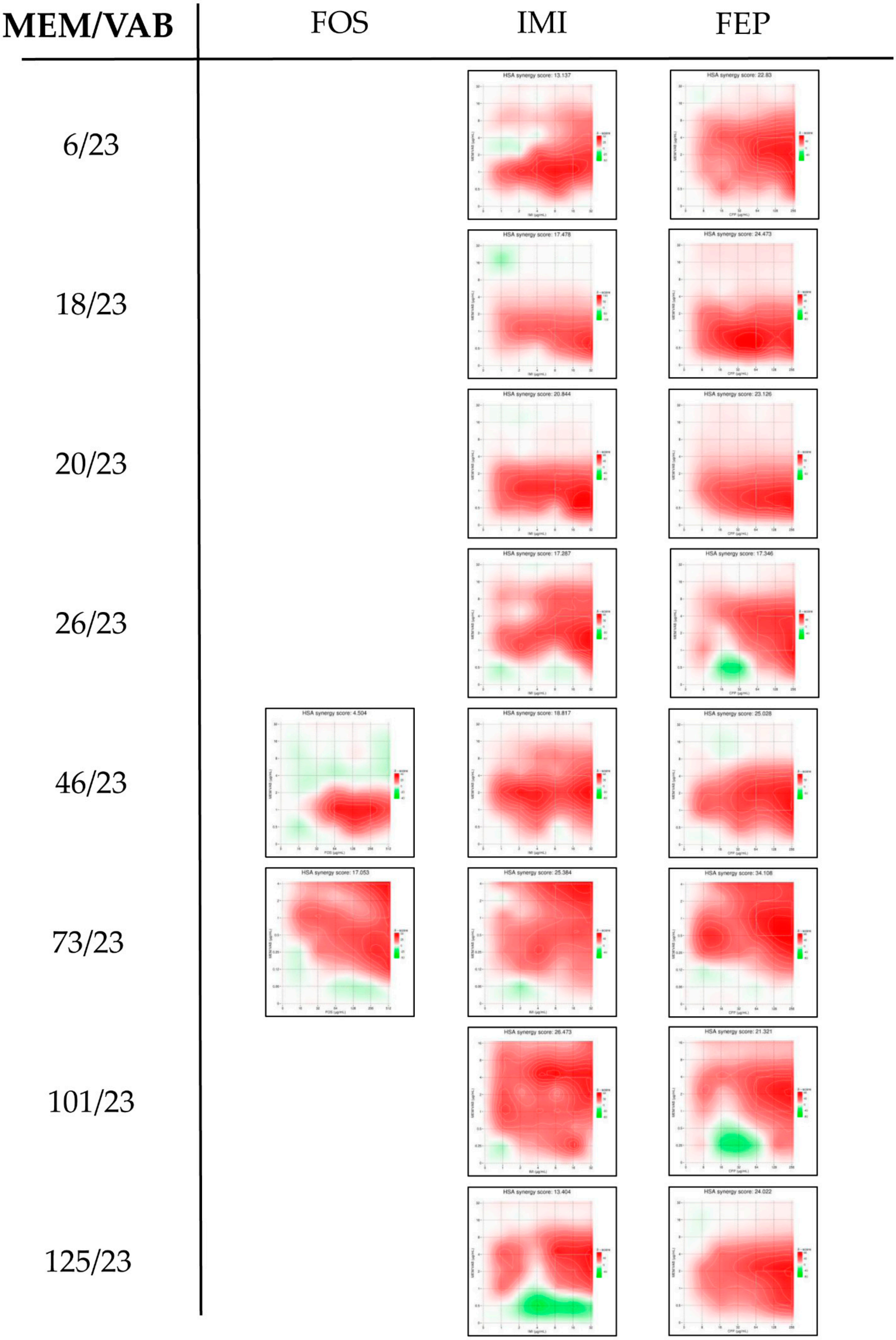
MEM/VAB
FOS
IMI
FEP
6/23
18/23
20/23
26/23
46/23
73/23
101/23
125/23
HSA synergy score: 13.137
HSA synergy score: 22.83
HSA synergy score: 17.478
HSA synergy score: 24.473
HSA synergy score: 20.844
HSA synergy score: 23.126
HSA synergy score: 17.287
HSA synergy score: 17.346
HSA synergy score: 4.504
HSA synergy score: 18.817
HSA synergy score: 25.028
HSA synergy score: 17.053
HSA synergy score: 25.384
HSA synergy score: 34.108
HSA synergy score: 26.473
HSA synergy score: 21.321
HSA synergy score: 13.404
HSA synergy score: 24.022

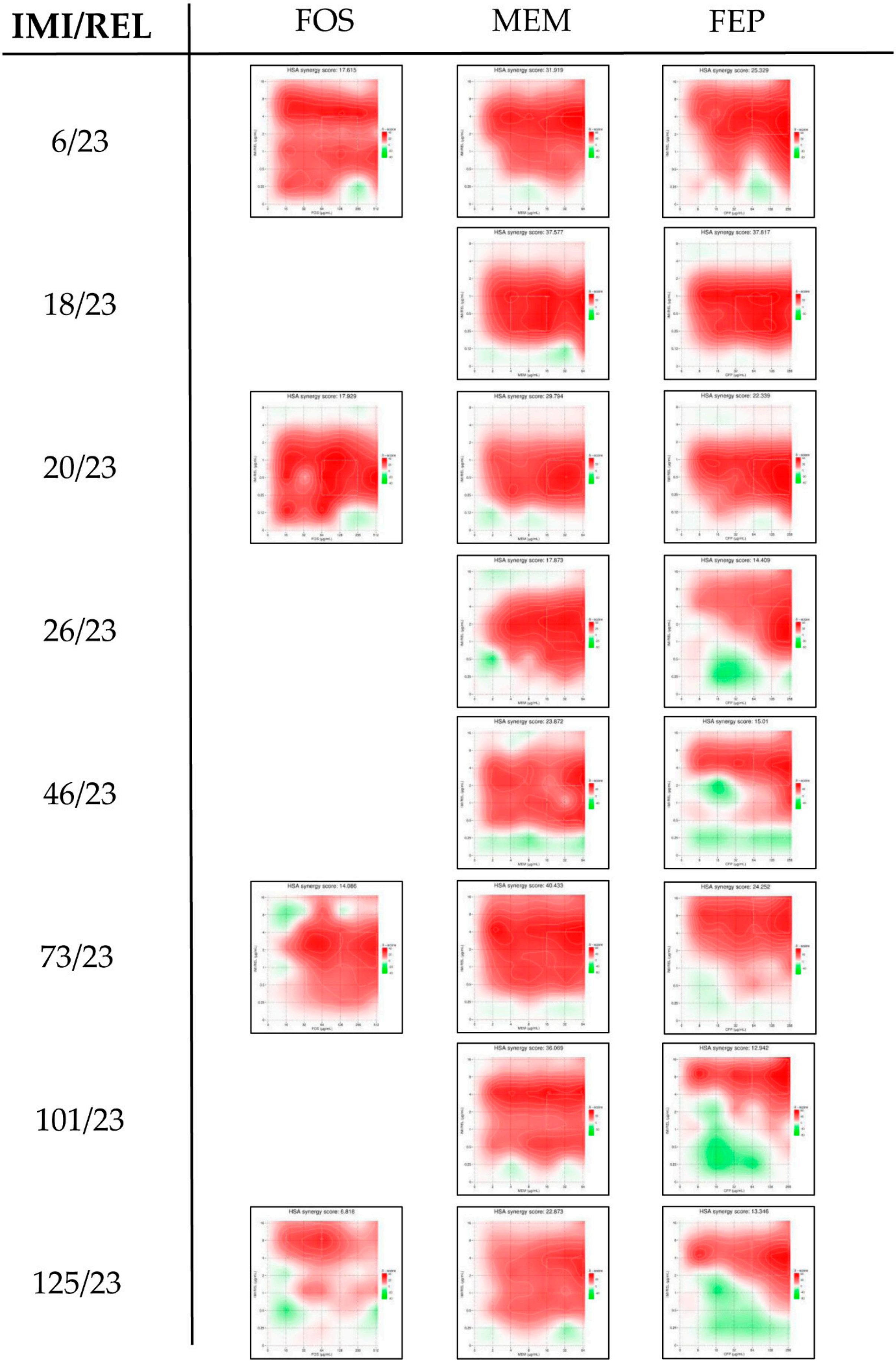
IMI/REL
FOS
MEM
FEP
6/23
18/23
20/23
26/23
46/23
73/23
101/23
125/23
HSA synergy score: 17.615
HSA synergy score: 31.919
HSA synergy score: 25.329
HSA synergy score: 37.577
HSA synergy score: 37.817
HSA synergy score: 17.929
HSA synergy score: 29.794
HSA synergy score: 22.339
HSA synergy score: 17.873
HSA synergy score: 14.409
HSA synergy score: 23.872
HSA synergy score: 15.01
HSA synergy score: 14.086
HSA synergy score: 40.433
HSA synergy score: 24.252
HSA synergy score: 36.069
HSA synergy score: 12.942
HSA synergy score: 6.818
HSA synergy score: 22.873
HSA synergy score: 13.346

Table S1. Reads generated per samples and reads classified.

Sample	Reads per sample	Reads Classified
**6/23**	2325131	2102833
**18/23**	2733905	2495018
**20/23**	2058891	1858387
**26/23**	2048332	1865277
**46/23**	1989185	1812465
**73/23**	2246852	2051487
**79/23**	1899384	1725200
**80/23**	2018461	1818259
**97/23**	1987342	1816222
**101/23**	1294115	1174840
**125/23**	2014697	1821515
**140/23**	1868209	1701756

Table S2. Anti-microbial resistance genes detected in *Klebsiella pneumoniae* isolates.

	**Isolates code**											
**Resistance genes**	**6/23**	**18/23**	**20/23**	**26/23**	**46/23**	**73/23**	**79/23**	**80/23**	**97/23**	**101/23**	**125/23**	**140/23**
aadA2	+	+	+	+	+					+	+	
aph(3')-Ia	+	+	+	+	+			+		+	+	
aph(6)-Id						+		+	+			
aac(6')-Ib-cr						+		+	+			
aph(3'')-Ib						+		+	+			
ant(2'')-Ia							+					
ant(3'')-Ia							+	+				
aadA1							+	+				
aac(3)-IIa									+			
armA												+
blaKPC-3	+	+	+	+	+	+	+	+	+	+	+	+
blaOXA-1						+			+			
blaOXA-9	+			+	+	+		+		+	+	+
blaOXA-10								+				
blaSHV-28								+				
blaSHV-100						+		+	+			+
blaSHV-106							+					
blaSHV-158	+	+	+	+	+						+	
blaSHV-159	+		+		+					+	+	
blaSHV-182				+						+		
blaTEM-1A	+	+		+				+		+	+	+
blaTEM-1B						+			+		+	
blaTEM-57					+							
blaCTX-M-15						+		+	+			
blaCMY-16								+				
fosA	+	+	+	+	+	+	+	+	+	+	+	+
fosA5	+	+	+	+	+		+			+	+	+
fosA6						+		+	+			
mph(A)	+	+	+	+	+					+	+	
catA1	+	+	+	+	+		+			+	+	

cmlA1								+				
floR								+				
qnrB1						+			+			
OqxA	+	+	+	+	+		+	+	+	+	+	+
OqxB	+	+	+	+	+		+	+	+	+	+	+
ARR-2								+				
sul1	+	+	+	+	+		+	+		+	+	+
sul2						+		+	+			
tet(A)								+				
dfrA5												+
dfrA12	+	+	+	+	+					+	+	
dfrA14						+		+	+			

Table S3. Plasmids, mutations and predicted mobility detected in *Klebsiella pneumoniae* isolates.

Isolate	Replicon	Mutations involved in carbapenemes resistance		Carbapenemase genes	Predicted Mobility
		ompK36	***ompK37***		
**6/23**	IncFIB(K)<br>IncFIB(pQil)<br>IncFII(K)<br>IncX3	A217S	I70M; I128M; N230G; T234H	*blaKPC-3, blaSHV-182, blaOXA-9, blaTEM-1A*	Conjugative
**18/23**	IncFIB(K)<br>IncFIB(pQil)<br>IncFII(K)<br>IncX3	A217S	I70M; I128M; N230G; T234H; E244D	*blaKPC-3, blaSHV-182*	Conjugative
**20/23**	IncFIB(K)<br>IncFIB(pQil)<br>IncX3	A217S	I70M; I128M; N230G; E244D	*blaKPC-19, blaSHV-182*	Conjugative
**26/23**	IncFIB(K)<br>IncFIB(pQil)<br>IncFII(K)<br>IncX3	A217S	I70M; I128M; N230G; M233Q; Q235Y; R239K; E244D	*blaKPC-3, blaSHV-182, blaOXA-9, blaTEM-1A*	Conjugative
**46/23**	IncFIB(K)<br>IncFIB(pQil)<br>IncFII(K)<br>IncX3	A217S	I70M; I128M; N230G	*blaKPC-3, blaSHV-182, blaOXA-9, blaTEM-1A*	Conjugative
**73/23**	IncFIB(K)<br>IncFIB(pQil)<br>IncFII(K)	A217S	I70M; I128M; N230G; E244D	*blaKPC-3, blaSHV-106, blaOXA-9, blaOXA-1, blaCTX-M-15*	Conjugative
**79/23**	ColRNAI, Col156, IncFIB, IncHI1B(pNDM-MAR)	A217S; N218H	I70M; I128M	*blaKPC-3, blaSHV-106*	Conjugative
**80/23**	IncC<br>IncFIB(K)<br>IncFIB(pQil)<br>IncFII(K)		I70M; I128M; N230G; T234H	*blaKPC-3, blaSHV-106, blaOXA-9, blaOXA-10, blaTEM-1A, blaCTX-M-15, blaCMY-16*	Conjugative
**97/23**	IncFIB(K)<br>IncFIB(pQil)<br>IncFII(K)		I70M; I128M; N230G; Q235Y	*blaKPC-3, blaSHV-106, blaOXA-1, blaTEM-1B, blaCTX-M-15*	Conjugative
**101/23**	IncFIB(K)<br>IncFIB(pQil)<br>IncFII(K)<br>IncX3	A217S	I70M; I128M; N230G; T234H	*blaKPC-3, blaSHV-182, blaOXA-9, blaTEM-1A*	Conjugative

**125/23**	IncFIB(K)<br>IncFIB(pQil)<br>IncFII(K)<br>IncX3	A217S	I70M; I128M; N230G; M233Q; T234H; Q235Y	*blaKPC-3, blaSHV-182, blaOXA-9, blaTEM-1A*	Conjugative
**140/23**	Col440II<br>ColRNAI<br>IncFIB(K)<br>IncFIB(pQil)	A217S; N218H	I70M; I128M	*blaKPC-3, blaSHV-106, blaOXA-9, blaTEM-1A*	Conjugative

Table S4. Number of total and unique genes in each sample.

Isolate	Sequence types	N. of genes	N. of unique genes
**6/23**	512	5.245	2
**18/23**	512	5.226	8
**20/23**	512	5.213	4
**26/23**	512	5.243	2
**46/23**	512	5.246	0
**73/23**	307	5.228	78
**79/23**	101	5.325	349
**80/23**	307	5.263	199
**97/23**	307	5.111	19
**101/23**	512	5.298	4
**125/23**	512	5.300	3
**140/23**	101	5.142	42

Table S5. Virulence factor genes detected in *Klebsiella pneumoniae* isolates.

**Isolate**	**Virulence factors gene**						**Other biofilm formation related genes**
	**Aerobactin**	**Enterobactin**	**Capsule Production**	**Mucoid phenotype**	**Fimbriae**	**Phagocytosis Inibition**	
**6/23**	*iutA*	*entA, entB, entC, entD, entE, entF*	*galF*	*rpmA*	*fimA, fimC, fimD, fimF, fimG, fimH, mrkD*	*acrA, acrB*	*treC, sdiA, luxS, tqsA, pgaABC, allS, lsrCD*
**18/23**	*iutA*	*entA, entB, entC, entD, entE, entF*	*galF*	*rpmA*	*fimA, fimC, fimD, fimF, fimG, fimH, mrkD*	*acrA, acrB*	*treC, sdiA, luxS, tqsA, pgaABC, allS, lsrCD*
**20/23**	*iutA*	*entA, entB, entC, entD, entE, entF*	*galF*	*rpmA*	*fimA, fimC, fimD, fimF, fimG, fimH, mrkD*	*acrA, acrB*	*treC, sdiA, luxS, tqsA, pgaABC, allS, lsrCD*
**26/23**	*iutA*	*entA, entB, entC, entD, entE, entF*	*galF*	*rpmA*	*fimA, fimC, fimD, fimF, fimG, fimH, mrkD*	*acrA, acrB*	*treC, sdiA, luxS, tqsA, pgaABC, allS, lsrCD*
**46/23**	*iutA*	*entA, entB, entC, entD, entE, entF*	*galF*	*rpmA*	*fimA, fimC, fimD, fimF, fimG, fimH, mrkD*	*acrA, acrB*	*treC, sdiA, luxS, tqsA, pgaABC, allS, lsrCD*
**73/23**	*iutA*	*entA, entB, entC, entD, entE, entF*	*galF*	*rpmA*	*fimA, fimC, fimD, fimF, fimG, fimH, mrkD*	*acrA, acrB*	*treC, sdiA, luxS, tqsA, pgaABC, allS, lsrCD*
**79/23**	*iucA, iucB, iucC, iucD, iutA*	*entA, entB, entC, entE, entF*	*galF*	*rpmA*	*fimA, fimC, fimD, fimF, fimG, fimH,*	*acrA, acrB*	*wcaJ, treC, sdiA, luxS, tqsA, pgaABC, allS, lsrCD*

					mrkD		
**80/23**	*iutA*	*entA, entB, entC, entD, entE, entF*	*galF*	*rpmA*	*fimA, fimC, fimD, fimF, fimG, fimH, mrkD*	*acrA, acrB*	*treC, sdiA, luxS, tqsA, pgaABC, allS, lsrCD*
**97/23**	*iutA*	*entA, entB, entC, entD, entE, entF*	*galF*	*rpmA*	*fimA, fimC, fimD, fimF, fimG, fimH, mrkD*	*acrA, acrB*	*treC, sdiA, luxS, tqsA, pgaABC, allS, lsrCD*
**101/23**	*iutA*	*entA, entB, entC, entD, entE, entF*	*galF*	*rpmA*	*fimA, fimC, fimD, fimF, fimG, fimH, mrkD*	*acrA, acrB*	*treC, sdiA, luxS, tqsA, pgaABC, allS, lsrCD*
**125/23**	*iutA*	*entA, entB, entC, entD, entE, entF*	*galF*	*rpmA*	*fimA, fimC, fimD, fimF, fimG, fimH, mrkD*	*acrA, acrB*	*treC, sdiA, luxS, tqsA, pgaABC, allS, lsrCD*
**140/23**	*iutA*	*entA, entB, entC, entE, entF*	*galF*	*rpmA*	*fimA, fimC, fimD, fimF, fimG, fimH, mrkD*	*acrA, acrB*	*wcaJ, treC, sdiA, luxS, tqsA, pgaABC, allS, lsrCD*

Table S6. Comparison of clinical parameters and isolate microbiological features between surviving and deceased patients (N = 12). Categorical data are presented as counts and percentages, while continuous data are shown as geometric means (for planktonic MICs) or arithmetic means ± standard deviation (BEC90 values). Missing MIC values were excluded from the geometric mean calculations. Biofilm eradication measurements were performed only on strong biofilm-producing strains (n = 5 in the survived group and n = 2 in the deceased group), with ambiguous non-linear regression curves/values excluded from the analysis. All planktonic MIC geometric means were calculated after log_2 transformation and subsequent back-transformation. Statistically significant differences ($p < 0.05$) are highlighted where applicable.

**Clinical parameter / isolate feature**	**Survived (n = 7)**	**Dead (n = 5)**	**p-value**
**Catheter removal (Source control)**	7 (100%)	1 (20%)	0.010
**Septic shock**	0 (100%)	2 (40%)	0.152
**BL-BLICs treatment**	5 (70%)	4 (80%)	1
**Predominant clones (ST)**	5 ST512 (70%),<br>2 ST307 (30%),<br>0 ST101 (0%)	2 ST512 (40%),<br>1 ST307 (20%),<br>2 ST101 (40%)	0.558
**High biofilm production**	6 (86%)	2 (40%)	0.222
**Geometric mean planktonic MIC to BL-BLICs (mg/L)**	CAZ/AVI: 2.44/4[#]<br>MEM/VAB: 0.23/8*<br>IMI/REL: 0.19/4*[§]	CAZ/AVI: 4.59/4[#]<br>MEM/VAB: 0.44/8*<br>IMI/REL: 0.87/4[§]	# 0.02<br>> 0.05<br>§ 0.008
**Mean biofilm eradication conc. (XTT BEC90, mg/L ± SD)**	CAZ/AVI: 48 ± 25.44<br>MEM/VAB: 26.72 ± 12.77<br>IMI/REL: 12.37 ± 4.33	CAZ/AVI: 67.59 ± 5.08<br>MEM/VAB: 19.22 ± 1.92<br>IMI/REL: 26.46 ± 9.29	> 0.05

*For all geometric mean calculations, left-censored MIC values reported with a "less than or equal to" sign (≤) were conventionally assigned to the next lower dilution (≤0.5 mg/L was computed as 0.25 mg/L, and ≤0.25 mg/L as 0.125 mg/L). Abbreviations: BL-BLICs, beta-lactam/beta-lactamase inhibitor combinations; CAZ/AVI, ceftazidime/avibactam; MEM/VAB, meropenem/vaborbactam; IMI/REL, imipenem/relebactam; ST, sequence type; MIC, minimum inhibitory concentration; BEC90, 90% biofilm eradication concentration; SD, standard deviation.
